# Complexity of the neutrophil transcriptome in early and severe rheumatoid arthritis: a role for microRNAs?

**DOI:** 10.1093/jleuko/qiaf090

**Published:** 2025-06-18

**Authors:** Michele Fresneda Alarcon, Genna Ali Abdullah, John Alexander Beggs, Isobel Kynoch, Andrew Sellin, Andrew Cross, Sam Haldenby, Philipp Antczak, Eva Caamaño Gutiérrez, Helen Louise Wright

**Affiliations:** Institute of Life Course and Medical Sciences, University of Liverpool, William Henry Duncan Building, 6 West Derby Street, Liverpool L7 8TX, United Kingdom; Institute of Life Course and Medical Sciences, University of Liverpool, William Henry Duncan Building, 6 West Derby Street, Liverpool L7 8TX, United Kingdom; School of Biosciences, University of Liverpool, Life Sciences Building, Crown Street, Liverpool L69 7ZB, United Kingdom; School of Biosciences, University of Liverpool, Life Sciences Building, Crown Street, Liverpool L69 7ZB, United Kingdom; Institute of Life Course and Medical Sciences, University of Liverpool, William Henry Duncan Building, 6 West Derby Street, Liverpool L7 8TX, United Kingdom; Institute of Life Course and Medical Sciences, University of Liverpool, William Henry Duncan Building, 6 West Derby Street, Liverpool L7 8TX, United Kingdom; Centre for Genomic Research, University of Liverpool, Biosciences Building, Crown Street, Liverpool L69 7ZB, United Kingdom; Computational Biology Facility, University of Liverpool, LIV-SRF, MerseyBio, Crown Street, Liverpool L69 7ZB, United Kingdom; Centre for Molecular Medicine, University of Cologne, Robert-Koch-Str 21, 50931 Cologne, Germany; Computational Biology Facility, University of Liverpool, LIV-SRF, MerseyBio, Crown Street, Liverpool L69 7ZB, United Kingdom; Institute of Systems, Molecular and Integrative Biology, University of Liverpool, Biosciences Building, Crown Street, Liverpool L69 7ZB, United Kingdom; Institute of Life Course and Medical Sciences, University of Liverpool, William Henry Duncan Building, 6 West Derby Street, Liverpool L7 8TX, United Kingdom

**Keywords:** interferons, microRNA, neutrophils, rheumatoid arthritis, RNAseq

## Abstract

Neutrophils are innate immune cells that drive the progression of rheumatoid arthritis through the release of reactive oxygen species, neutrophil extracellular traps, and proteases that damage host tissues. Neutrophil activation is regulated, in part, by dynamic changes in gene expression. In this study, we have used RNAseq to measure the transcriptomes of neutrophils from people with severe, methotrexate-refractory rheumatoid arthritis and healthy controls. We identified a dynamic gene expression profile in people with severe rheumatoid arthritis. This is dominated by a type-I interferon-induced gene expression signature as well as activation of genes regulating neutrophil degranulation, neutrophil extracellular trap production, response to reactive oxygen species, and oxidative stress. While we did not detect significantly elevated levels of interferon-alpha in rheumatoid arthritis blood sera, we identified increased expression in rheumatoid arthritis neutrophils of miR-96-5p and miR-183-5p, which regulate activation of the interferon pathway as members of the miR-183C cluster. We also detected significantly elevated neutrophil extracellular trap debris in rheumatoid arthritis blood sera (*P* < 0.05). Using gene set variation analysis, we explored the heterogeneity of neutrophil gene expression in rheumatoid arthritis and identified subsets of patients with gene expression profiles reflecting enhanced neutrophil degranulation and cytotoxicity, tissue inflammation, or activation by interferons. Comparison with published single-cell RNAseq datasets identified rheumatoid arthritis transcriptomes where neutrophils were polarized by genes relating to early or late cell maturity, with significant genes in each polarized state being regulated by miR-146a-5p, miR-155-5p, miR-183-5p, or miR-96-5p. Overall, our study demonstrates the heterogeneity of the rheumatoid arthritis neutrophil transcriptome and proposes microRNA-driven mechanisms for regulating the activated neutrophil phenotype in rheumatoid arthritis.

## Teaser

Rheumatoid arthritis neutrophils have heterogeneous gene expression profiles associated with neutrophil polarization and activation states, which we propose are regulated by microRNAs.

## 1. Introduction

Rheumatoid arthritis (RA) is a chronic autoimmune disorder characterized by persistent inflammation and the progressive destruction of joint tissues. While the exact etiology of RA remains unclear, it is well understood that the disease involves a complex interplay of genetic, environmental, and immunological factors.^1^ Among the key players in the pathogenesis of RA are neutrophils, which are the most abundant type of blood leukocyte in humans and play a crucial role in the innate immune response.^2,3^ Neutrophils are traditionally recognized for their role in the rapid response to infections, being the first immune cells to arrive at sites of inflammation. They can engulf and destroy pathogens through phagocytosis, release antimicrobial proteases, and generate neutrophil extracellular traps (NETs) to immobilize and kill microbes.^1^ However, in autoimmune diseases like RA, the dysregulated activation of neutrophils contributes to the pathology of the disease rather than resolving any infection.^1,4^

In RA, neutrophils are excessively activated and accumulate in the synovial fluid and tissue of affected joints.^5,6^ This accumulation not only reflects an increased influx but also decreased apoptosis, leading to prolonged survival of these cells in the joint space.^7,8^ Activated neutrophils in the RA synovium release a range of proinflammatory mediators, including cytokines interleukin-1 (IL-1) and tumor necrosis factor-alpha (TNF-α) as well as a number of chemokines, which further amplify the inflammatory response.^9,10^ Additionally, they produce reactive oxygen species (ROS) and proteolytic enzymes including collagenase, contributing to tissue damage and the erosion of cartilage and bone.^3,6,11–13^

Recent advancements in molecular biology and genomics have shed light on the gene expression profiles of neutrophils in RA, particularly during inflammation. Gene expression profiling studies have shown that neutrophils from people with RA exhibit distinct transcriptional signatures compared with those from healthy individuals.^14,15^ These signatures are characterized by the upregulation of genes involved in inflammatory responses, migration, and survival.^16^ For instance, genes encoding components of the NADPH oxidase complex, which plays a critical role in the production of ROS, are often upregulated in RA neutrophils. Furthermore, the interaction between neutrophils and other immune cells, such as T cells and macrophages, is also influenced by their gene expression. The expression of surface molecules that modulate immune cell interactions, such as adhesion molecules and antigen-presenting molecules, is altered in RA neutrophils.^16^ This can affect the recruitment and activation of other immune cells, contributing to the perpetuation of the inflammatory process.^17–19^

Another important aspect of RA neutrophils is their role in forming NETs, which are formed from externalized DNA, histones, and neutrophil-derived proteins. In RA, the formation of NETs is thought to contribute to the autoimmune response by exposing intracellular antigens to the immune system.^20,21^ Studies have shown that NETs are abundant in the synovial fluid of RA patients and may be involved in the formation of autoantibodies typical of RA, such as anticitrullinated protein antibodies.^6,20,22^ We have also recently identified a subpopulation of DEspR positive, neutrophil extracellular trap (NET)-forming neutrophils in RA blood smears.^23^

In this study, we aim to further delineate the heterogeneity of gene expression profile of RA neutrophils and propose inflammatory factors regulating gene expression and production of proinflammatory molecules by neutrophils. We also demonstrate altered expression of microRNAs (miRNAs) involved in regulating neutrophil messenger RNA (mRNA) expression. Understanding the specific pathways and molecular mechanisms underlying the abnormal behavior of neutrophils in RA could open new avenues for targeted therapies, potentially leading to better management and treatment of this debilitating condition.

## 2. Methods

### 2.1 Ethics and patients

This study was approved by the University of Liverpool Central University Research Ethics Committee C for healthy controls (Ref: 1672) and NRES Committee North West (Greater Manchester West, United Kingdom) for people with RA (Ref: 11/NW/0206). All participants gave written, informed consent in accordance with the Declaration of Helsinki. All patients with RA fulfilled the American College of Rheumatology 2010 criteria for the diagnosis of RA. Disease activity was recorded using 28-joint disease activity score (DAS28), and improvements in disease activity were measured using European Alliance of Associations for Rheumatology criteria.^24^ Th early RA (ERA) cohort comprised newly diagnosed patients prior to commencing disease-modifying antirheumatic drug (DMARD) therapy. Severe RA (SRA) patients were people with a DAS28 score ≥5.1 recruited prior to commencing anti-TNF (TNFi therapy). Healthy controls were recruited from staff at the University of Liverpool. All participants were over the age of 18 yr and free of infection. Participant demographics are shown in Table 1.

**Table 1.** Participant demographics.

	ERA	SRA	Healthy controls
*n*	13	65	11
Age (mean ± SD)	52.52 (±12.99)	52.79 (±10.64)	47.80 (±10.16)
Sex (% female)	62	80	45
DAS28 (mean ± SD)	5.19 (±1.43)	5.92 (±0.82)	N/A

Shows participant demographics across the 3 groups, ERA, SRA, and healthy controls.

### 2.2 Neutrophil isolation and culture

Neutrophils were isolated from heparinized peripheral blood using HetaSep and Ficoll Paque as previously described.^25^ Contaminating erythrocytes were lysed using ammonium chloride lysis buffer. Neutrophils were resuspended in Roswell Park Memorial Institute 1640 media (Life Technologies) containing L-glutamine (2 mM) and Hepes (25 mM) at a concentration of 5 × 10^6^/mL unless otherwise stated. Neutrophil purity was routinely >97% and viability >98%.

### 2.3 RNA isolation

RNA was isolated from 10^7^ neutrophils using an optimized Trizol-chloroform protocol (Life Technologies),^26^ precipitated in isopropanol and cleaned using the QIAGEN RNeasy (mRNA) or miRNeasy (miRNA) kit including a DNase digestion step. RNA was snap-frozen in liquid nitrogen and stored at −80 °C. Total RNA concentration and integrity were assessed using the Agilent 2100 Bioanalyzer RNA Nano or Pico chip. RNA integrity was routinely ≥7.0.

### 2.4 mRNA sequencing

Total RNA was enriched for mRNA using poly-A selection. For cohort 1, 50-bp single-end read libraries were sequenced on the Illumina HiSeq 2000 platform. For cohort 2, 100-bp paired-end read libraries were sequenced on the DNBseq platform. Reads were mapped to the human genome (hg38) using HISAT2.^27^ Read counts were generated using featureCounts, which is part of the Rsubread package (v2.0.1)^28^ for R (v4.0.2).^29^ Statistical analysis of gene counts was carried out using edgeR generalized linear modeling (v3.28.1)^30^ with the “intercept” representing the mean gene expression for the healthy control (HC) group and coefficients in the model representing ERA and SRA groups. Batch correction was built into the model design, and batch-corrected gene counts (cpm) were produced using limma.^31^ Differential expression testing was performed using genewise negative binomial generalized linear model test, with a false discovery rate correction to *P*-values. The raw sequencing data reported in this manuscript have been deposited in the NCBI Gene Expression Omnibus (GEO)^32^ and are accessible through GEO Series accession numbers GSE274598 (https://www.ncbi.nlm.nih.gov/geo/query/acc.cgi?acc=GSE274598) and GSE274996 (https://www.ncbi.nlm.nih.gov/geo/query/acc.cgi).

### 2.5 miRNA sequencing

Total RNA was size selected (60 to 300 nt), decapped, and sequenced on the Illumina NovaSeq platform. miRNAs reads were processed with miRge3.0,^33^ which aligned reads to the human miRBase database (version 22)^34^ with Bowtie.^35^ Statistical analysis of miRNAs was performed using DESeq2 (contrasts RA vs HC)^36^ in R (v4.0.2),^29^ applying a Benjamini–Hochberg (BH) false discovery rate (FDR) correction to *P*-values. The processed miRNA data reported in this manuscript can be accessed via FigShare at: 10.6084/m9.figshare.27136062.

### 2.6 Bioinformatics and statistical analysis

Gene ontology enrichment analysis of genes with 1.5-fold increased expression between RA and HC was carried out using R package clusterProfiler against the genome-wide annotation for human (org.Hs.eg.db)^37,38^ and included overrepresentation searches in the Reactome database.^39^ Gene set enrichment analysis was carried out in R using the CRAN package tmod^40^ and based on a modular framework for classifying blood genomics studies.^41^ Gene expression network analysis was carried out using ARACNE2^42^ filtering on a mutual information (MI) threshold of 0.5 (*P* = 10^−20^). Gene networks were visualized using Cytoscape^43^ using GLay clustering within the clustermaker2 package.^44^ Genes within each cluster were analyzed for overrepresentation of Gene Ontology categories using BINGO.^45^ Functional enrichment analysis of genes with increased or decreased expression between RA and HC, and miRNA:mRNA target enrichment analysis, was complemented with Ingenuity Pathway Analysis (https://digitalinsights.qiagen.com/IPA)^46^ applying a 1.5-fold change in gene expression cutoff and a BH correction to *P*-values for canonical pathway analysis, using the Ingenuity Knowledge Base as background. Gene set variation analysis (GSVA) was performed using the GSVA package^47^ against the molecular signatures database (MSigDB)^48^ in October 2023. Datasets used were C5 Gene Ontology Biological Processes (GOBPs), Human Phenotype Ontology, C7 immunologic signatures, and C8 cell type signature gene sets.

Differential gene expression analysis of transcriptomics data is detailed above. Statistical analysis of experimental data was performed using R (v4.0.2).^29^ The Shapiro–Wilk test was complemented with QQ plot analysis to determine normality. Then univariate analysis was carried out by the Student's *t*-test or Wilcoxon test as appropriate. Correlation test was performed in R (v4.0.2), and a significant correlation was considered when the *P*-value post FDR adjustment (BH) was <0.05.

### 2.7 Protein extraction and western blotting

Neutrophil proteins were extracted from Trizol lysates using the manufacturer's instructions into Laemmli buffer containing 1:1 3% SDS and 9 M urea without bromophenol blue. Protein concentrations were measured using the bicinchoninic acid assay (Pierce) and normalized across samples before loading onto sodium dodecyl sulfate polyacrylamide gel (SDS-PAGE) gel. Protein samples were separated by SDS-PAGE using a 12% gel and transferred onto polyvinylidene fluoride membrane (Merck). Primary antibodies were as follows: catalase (CAT; 1:1,000 Abcam), glutathione peroxidase (GPx; 1:1,000 Abcam), and glyceraldehyde-3-phosphate dehydrogenase (1:1,000, Merck). Secondary antibodies were antirabbit IgG-specific horse radish peroxidase (HRP)-linked (1:10,000, GE Healthcare) and antimouse IgG-specific HRP-linked (1:10,000, Abcam). Bound antibodies were detected using the ECL system (Merck) and carefully exposed film to avoid saturation.

### 2.8 Enzyme-linked immunosorbent assays

Serum samples were collected using Z-clot serum vacutainers, and aliquots snap-frozen in liquid nitrogen before storing at −80 °C. Sera were clarified by centrifugation at 1000 × *g* for 15 min prior to performing ELISA. Interferon-α (IFN-α) concentration was measured in serum using the VeriKine high-sensitivity IFN-α (all subtypes) ELISA Kit (PBL assay science) using the manufacturer's instructions. The presence of NETs in sera was measured using the capture antibody from the human myeloperoxidase ELISA Kit (Abcam) and the anti-DNA detection antibody from the Cell Death Detection ELISAplus (Merck). The standard curve was comprised of NETs produced in vitro by human neutrophils in response to phorbol 12-myristate 13-acetate, as previously described.^49,50^ Enzyme-linked immunosorbent assay (ELISA) analyte concentrations were determined by 4-parameter logistic regression with reference to the assay-specific standard curve.

### 2.9 Enzyme activity assays

Superoxide dismutase (SOD) activity was measured using the SOD activity colorimetric assay (Abcam). CAT activity was measured using the CAT activity fluorometric assay (Abcam). GPx activity was measured using the GPx activity colorimetric assay (Abcam). For all assays, neutrophils (2 × 10^6^) were washed with ice-cold phosphate buffered saline, centrifuged at 1,000 × *g* for 3 min, and pellets lysed in 200 µL ice-cold assay buffer. Cell lysates were centrifuged at 10,000 × *g* for 15 min at 4 °C, and supernatant pipetted into a clean tube before snap freezing in liquid nitrogen and storing at −80 °C prior to analysis. Enzyme activity assays were performed using the manufacturer’s instructions, and enzyme activity was calculated from either a standard curve (GPx and CAT assays) or expressed as a percentage inhibition rate based on positive and negative control wells (SOD assay) as described in the manufacturer’s instructions.

## 3. Results

### 3.1 Neutrophil gene expression in SRA is driven by IFNs

**Fig. 1.**
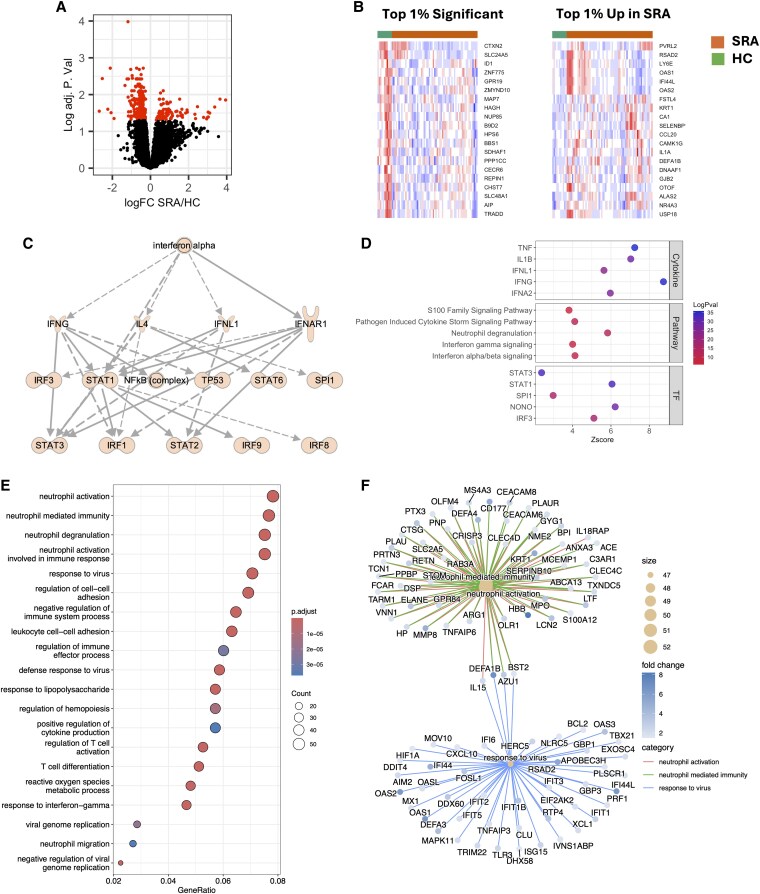
Transcriptomic analysis of SRA neutrophils. A) Volcano plot showing genes highlighted in red upregulated and downregulated in SRA compared with HC (adj. *P* < 0.05). B) Heatmaps showing top 1% significant and top 1% upregulated genes in SRA compared with HC. Orange bar indicates SRA samples, and green bar indicates HC samples. C) IPA prediction of IFN signaling network upregulated in SRA. D) Summary of IPA upstream analysis of cytokines, canonical pathways, and transcription factors (TFs) activated in SRA neutrophils. E) GOBP analysis of genes upregulated 1.5-fold in SRA neutrophils. F) Plot of genes common to GOBP ontologies relating to neutrophil-mediated immunity, neutrophil activation, and response to virus.

In our previous study, we identified that peripheral blood RA neutrophils have a different gene expression profile from healthy controls, driven typically by either a type-I IFN-induced signature or expression of genes relating to neutrophil granule proteases.^15^ For this study, we recruited a second, larger, cohort of Biologics-naïve RA patients with high disease activity (DAS28 > 5.1), termed SRA. We combined our 2 cohorts of SRA patients together (*n* = 65 in total) and compared gene expression with healthy control (HC, *n* = 11) neutrophil transcriptomes as well as an ERA (*n* = 13) cohort of newly diagnosed patients, pre-DMARD therapy. We determined that 387 genes were significantly different between SRA and HC neutrophils (FDR adj. *P* < 0.05, Fig. 1A). The top 1% of significantly different genes were all downregulated in SRA neutrophils compared with HC (ie higher in HC neutrophils, Fig. 1B). Functional overrepresentation analysis of significant genes using the Reactome database against total resources for *Homo sapiens* identified enrichment of signaling pathways for “transcriptional regulation by small RNAs” (*P* < 0.001) and “the AIM2 inflammasome “ (*P* < 0.01). SRA neutrophils showed high heterogeneity in the top 1% of upregulated genes (Fig. 1B), with distinct clusters of SRA patients with “high” or “low” IFN-response gene expression. Reactome analysis identified significant enrichment of signaling pathways from “IFN-α/beta signaling” (*P* < 0.001), “cytokine signaling in immune system” (*P* < 0.001), and “alpha-defensins” (*P* < 0.001).

In order to investigate the altered transcriptome of SRA neutrophils further, we used IPA to predict major signaling pathways regulating SRA neutrophil gene expression. As previously described, an IFN-α-regulated network of genes was the highest predicted interaction network by IPA (Fig. 1C, adj. *P* < 0.01). IFN-gamma and IFN-α signaling were highly predicted canonical pathways activated in SRA neutrophils (Fig. 1D, adj. *P* < 0.01). Other pathways significantly activated were neutrophil degranulation, pathogen-induced cytokine storm signaling, and S100 family signaling (Fig. 1D, adj. *P* < 0.01). Of note, the neutrophil degranulation canonical pathway is enriched with genes for granule proteins including *MPO*, *ELANE*, and lactoferrin (*LTF*), which are likely to be detected due to the presence of immature neutrophils rather than increased expression of granule protein genes.^14,51^ Further neutrophil-specific canonical pathways that were predicted to be activated in SRA were NET signaling, granulocyte adhesion and diapedesis, and transcriptional regulation of granulopoiesis phagosome formation (adj. *P* < 0.01). Several pathways regulating oxidative stress were also predicted to be activated. Upstream regulator cytokine analysis predicted regulation of SRA neutrophil gene expression by IFNs (IFNA1, IFNG, and IFNL1), IL-1β, and TNFα (Fig. 1D, adj. *P* < 0.01; Table S1). Predicted transcription factor activation included interferon regulatory factor 3 (IRF3), transcription factor PU.1 (SPI1), signal transducer and activator of transcription 1 (STAT1), signal transducer and activator of transcription 3 (STAT3), and non POU domain-containing octamer-binding protein (NONO) (Fig. 1D, adj. *P* < 0.01; Table S1). We complemented IPA analysis with Gene Ontology (GO) analysis accessed through the R package clusterProfiler. We confirmed HC vs SRA differences, relating to neutrophil activation, degranulation, and neutrophil-mediated immunity, as well as response to virus and cell–cell adhesion (Fig. 1E and F).

### 3.2 Neutrophil gene expression in treatment-naïve RA is similar to SRA

**Fig. 2.**
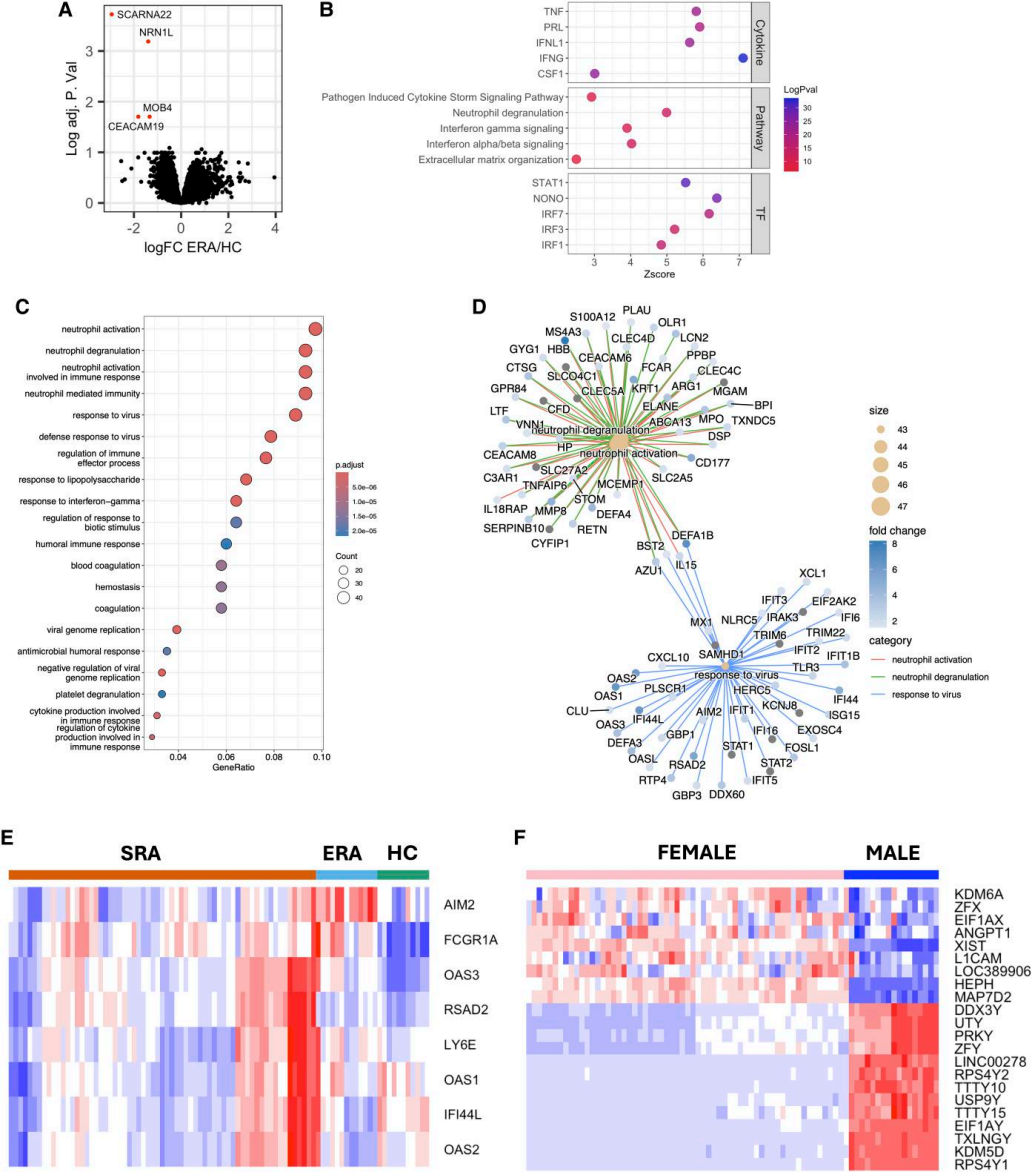
Transcriptomic analysis of ERA neutrophils. A) Volcano plot showing genes highlighted in red as being significantly upregulated in ERA compared with HC (adj. *P* < 0.05). B) Summary of IPA upstream analysis of cytokines, canonical pathways, and transcription factors (TFs) activated in ERA neutrophils. C) Gene ontology analysis of genes upregulated 1.5-fold in ERA neutrophils. D) Plot of genes common to GOBP ontologies relating to neutrophil degranulation, neutrophil activation, and response to virus. E) Heatmap of IFN-response gene expression in HC, ERA, and SRA neutrophils. F) Heatmap of sex-associated genes with differential expression between female and male study participants (adj. *P* < 0.05).

We next sought to determine whether RA neutrophil gene expression changes during the course of the disease. Only 4 genes were significantly different between ERA patients and HC (Fig. 2A, adj. *P* < 0.05), and all were lower in people with ERA. IPA revealed once again that the pathways upregulated in ERA neutrophils were IFN-α/beta signaling, IFN-gamma signaling, and neutrophil degranulation as well as pathogen-induced cytokine storm signaling and extracellular matrix organization (Fig. 2B, adj. *P* < 0.01). Upstream cytokine regulators of ERA gene expression were predicted to be IFNs gamma and lambda, TNF, prolactin, and colony stimulating factor 1 (Fig. 2B, adj. *P* < 0.01; Table S2). Similarly to SRA, the transcription factors predicted to be activated were IRF1, IRF3 IRF7, NONO, and STAT1 (Fig. 2B, adj. *P* < 0.01; Table S2). Gene ontology analysis of ERA neutrophil gene expression was surprisingly similar to SRA and enriched for genes regulating neutrophil activation and degranulation, response to virus, and regulation of immune effector process (Fig. 2C and D). Expression of IFN-response genes was similar in ERA and SRA, both of which were higher than in HC (Fig. 2E). This led us to conclude that the neutrophil gene expression signature is enhanced but not significantly changed when RA disease activity does not resolve with DMARDs. Preliminary proteomics analysis of SRA neutrophils (*n* = 3) confirmed the elevation of the type-I IFN response at the protein level, with expression of several type-I IFN-response proteins being >2-fold higher in SRA neutrophils compared with healthy controls (data not shown). Linear modeling of ERA and SRA neutrophil transcriptomes did not identify any genes with expression associated to the age of the study participants or disease activity score (DAS28) after *P*-value correction. However, 22 genes were differentially expressed between male and female participants (Fig. 2F). Of these genes, 13 were located on the Y-chromosome (*DDX3Y, PRKY, TTTY15, TXLNGY, USP9Y, UTY,* and *ZFY*) and expressed higher in neutrophil from male donors (adj. *P* < 0.05). Eight X-chromosome (*EIF1AX, HEPH, KDM6A, L1CAM, LOC389906, MAP7D2, XIST,* and *ZFX*) and 1 chromosome-8 gene (*ANGPT1*) were expressed higher in neutrophil from female donors (adj. *P* < 0.05). Analysis of the 2 sex-associated gene lists using Reactome revealed that both sets of genes were significantly enriched for “HDMs demethylate histones” (adj. *P* < 0.05) indicating male and female neutrophils may activate different enzymes to perform the same cellular functions.

**Fig. 3.**
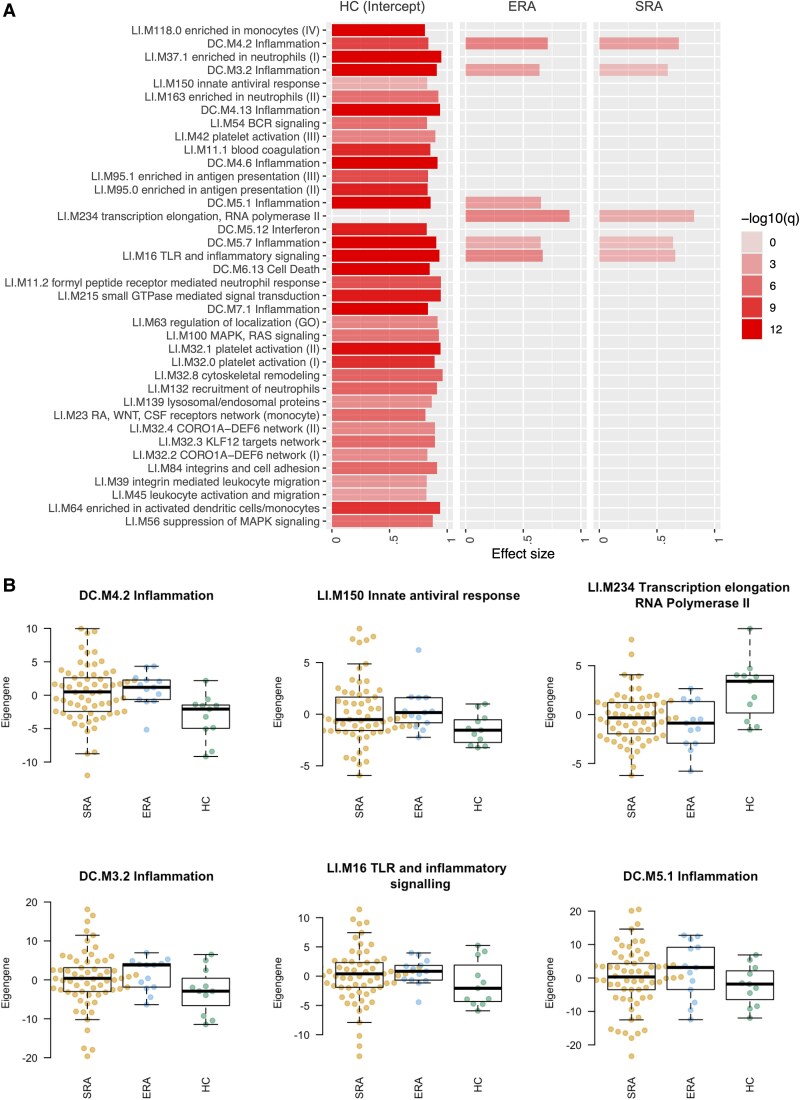
Gene set enrichment analysis of SRA, ERA, and HC neutrophils. A) Modular enrichment analysis (tmod) of genes expressed in SRA, ERA, and HC neutrophils (AUC > 0.8) using an intercept model. B) Eigengene expression in SRA, ERA, and HC neutrophils for selected modules relating to inflammation (DC.M4.2, DC.M3.2, and DC.M5.7), TLR activation (LI.M16), transcription elongation (LI.M234), and innate antiviral response (LI.M150).

We next used blood transcriptional modular enrichment analysis (R package tmod) to identify gene expression modules enriched in SRA, ERA, and HC neutrophils across our linear model. This modular analysis is based on signaling modules compiled from analysis of blood transcriptomes across a number of inflammatory conditions.^41^ Modules relating to neutrophils (LI.M37.1, LI.M163, LI.M11.2, and LI.M132), antigen presentation (LI.M95.0 and LI.M95.1), integrins (LI.M84 and LI.M39), and mitogen-activated protein kinase signaling (LI.M100 and LI.M56) were significantly enriched in healthy neutrophils (intercept) (Fig. 3A). Modules relating to inflammation (DC.M4.2, DC.M3.2, and DC.M5.7) and Toll-like receptor (TLR) activation (LI.M16) and transcription elongation (LI.M234) were further significantly enriched in SRA and ERA neutrophils compared with HC (Fig. 3A and B). Module DC.M5.1, inflammation was enriched in ERA neutrophils only.

### 3.3 Gene networks regulate RA neutrophil gene expression

**Fig. 4.**
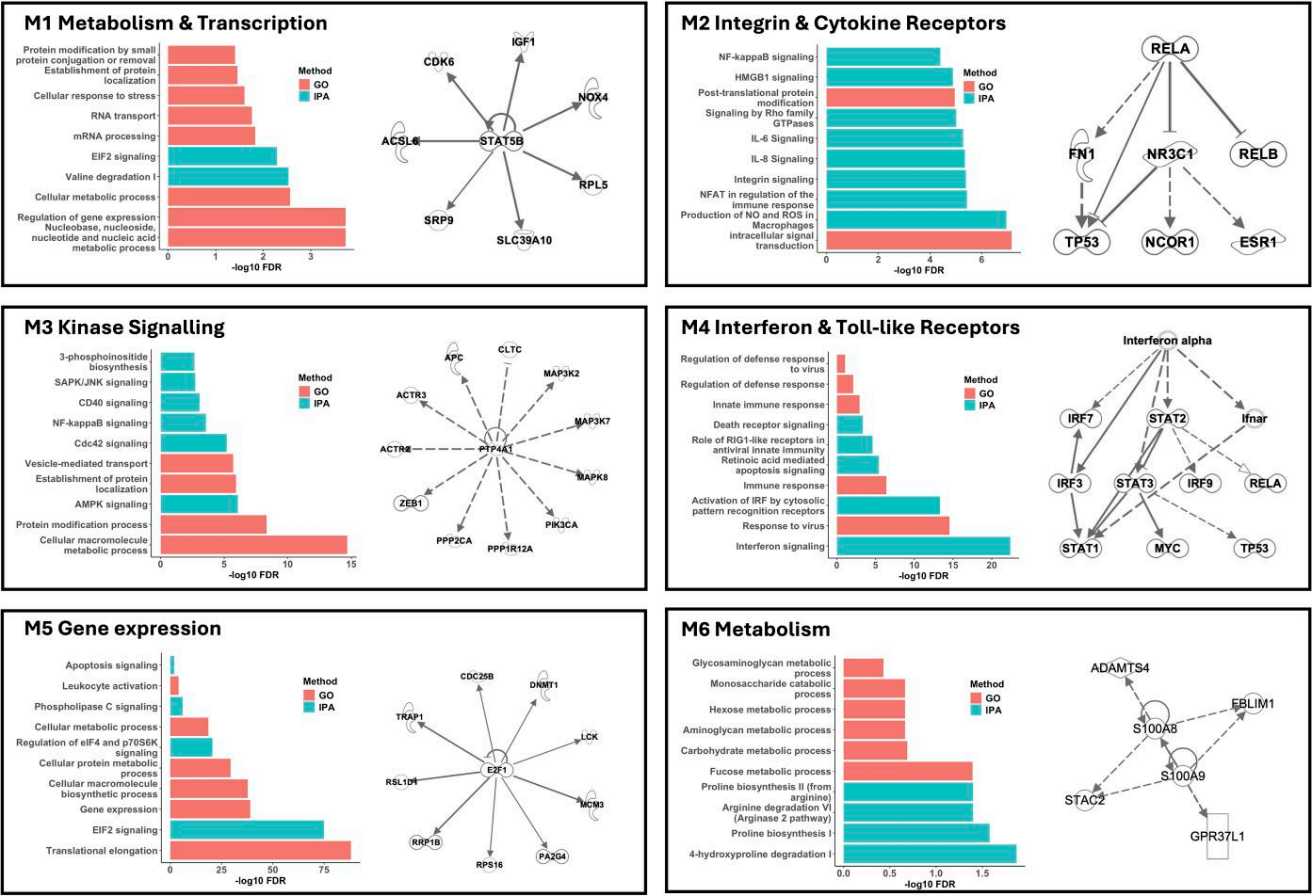
Modular gene network analysis identified 6 major gene networks in SRA neutrophils. Each panel shows reconstructed network numbered Mx, with bar chart showing gene ontologies (pink) and IPA canonical pathways (blue) activated within the gene network. Right panel in each module indicates upstream regulator “hub” genes within each module.

We next wanted to further investigate the regulation of gene networks in SRA neutrophils. To do this, we used ARACNE2 to reconstruct the gene expression networks in SRA neutrophils, applying a MI threshold of 0.5 (*P* < 10^−20^). Modules were reconstructed using Cytoscape and clustermaker^43,44^ and analyzed for gene ontology overrepresentation using the BINGO app^45^ and for canonical pathway enrichment using IPA. We identified 6 major modules of gene expression networks in SRA neutrophils (Fig. 4; Table S3). Module 1 (M1) was related to metabolism and transcription, incorporating essential cell functions such as regulation of transcription, protein modification, and localization, metabolic processing of nucleosides and amino acids, and cellular response to stress. M2 was regulated by integrins and cytokine receptors including IL-6 and IL-6 signaling, nuclear factor kappa-light-chain-enhancer of activated B cells (NF-κB), nuclear factor of activated T-cells, and high mobility group protein B1 (HMGB1) signaling, and signaling by Rho GTPases. M3 regulated kinase activation including 5' adenosine monophosphate (AMP)-activated protein kinase, stress-activated protein kinases/Jun amino-terminal kinases, Cdc42, and NF-κB as well as 3-phosphoinositide biosynthesis. M4 was a much smaller network of genes regulated by IFN and TLR receptor signaling. M5 was another module regulating gene expression, this time incorporating phospholipase C and apoptosis signaling as well as gene translation and metabolism of proteins. Finally, M6 contained a gene network regulating carbohydrate and amino acid metabolism.

### 3.4 NET debris is elevated in RA sera

**Fig. 5.**
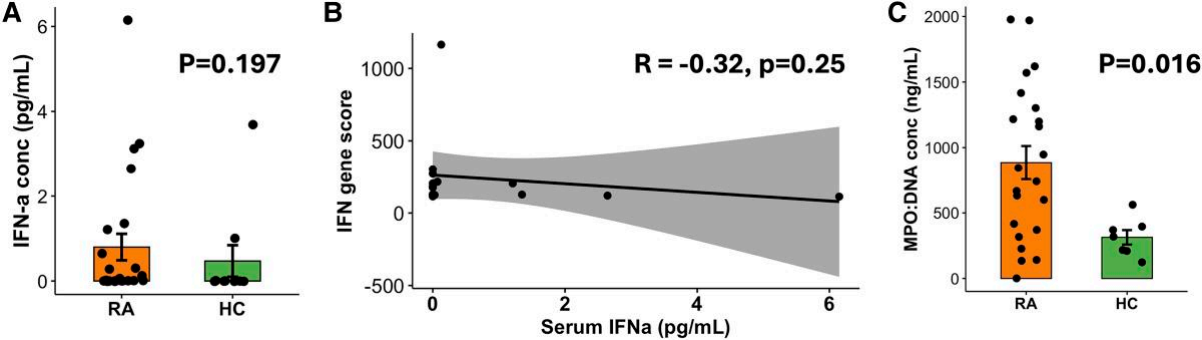
Serum inflammatory markers in people with SRA. A) Serum IFN-α (all types) concentrations measured by high-sensitivity ELISA in SRA (orange) and HC (green) sera. B) Correlation of IFN-α serum concentrations with average expression of IFN-response genes (IFN gene score) in neutrophils. C) Concentrations of NET debris (DNA:MPO complexes) in SRA (orange) and HC (green) sera (*P* < 0.05).

Our next aim was to validate the bioinformatics predictions and determine the factor(s) regulating neutrophil gene expression in SRA. We attempted to measure the level of IFN-α in SRA sera collected at the same time as the neutrophils we subjected to RNAseq. We were unable to detect any IFN-α in SRA or HC sera using commercial ELISA kits (data not shown). However, using a high-sensitivity IFN-α (all subtype) ELISA, we detected very low concentrations of IFN-α in 12 of 24 SRA sera assayed (mean 0.8 pg/mL) compared with 2 of 10 HC sera (mean 0.47 pg/mL). This difference was not statistically significant (Fig. 5A, *P* = 0.197), and the concentrations of IFN-α in SRA sera did not correlate with expression of IFN-response genes (Fig. 5B, *P* = 0.25). No expression of any IFN-α genes was detected in any neutrophil RNAseq samples (data not shown). We also performed a custom sandwich ELISA on SRA and HC sera to measure the presence of NET debris (MPO:DNA complexes), and this identified significantly higher levels of NET debris in RA sera compared with healthy controls (Fig. 5C, *P* < 0.05).

### 3.5 Antioxidant proteins are lower in RA neutrophils

**Fig. 6.**
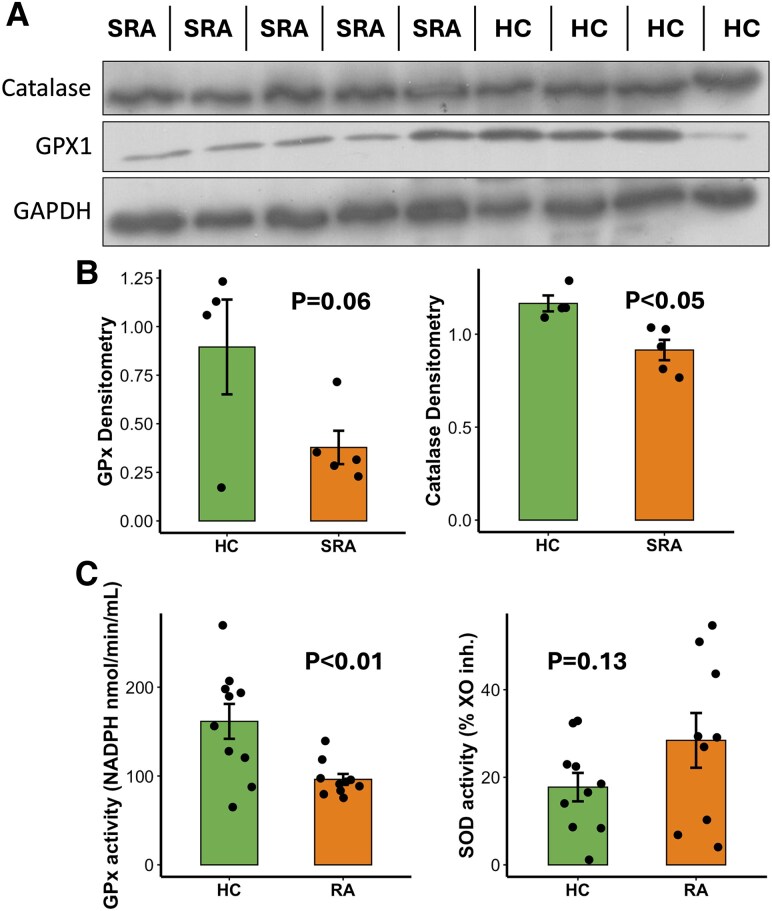
Antioxidant enzyme expression and activity in RA neutrophils. A) Protein levels measured by western blotting of CAT and GPx. B) Western blot of protein lysates from SRA (*n* = 5) and HC (*n* = 4). C) Enzyme activity levels of CAT, GPx, and SOD in SRA (orange) and HC (green) neutrophils.

We next investigated the role of oxidative stress in RA neutrophils, as several pathways relating to oxidative stress and response to ROS were identified by IPA in SRA and ERA neutrophils. Western blot analysis of neutrophil protein lysates showed lower expression of CAT (Fig. 6A and B, *P* < 0.05) and GPx (Fig. 6A and B, *P* < 0.06) in SRA neutrophils compared with HC. Enzyme activity assay of neutrophil cell lysates identified that GPx activity was lower in SRA neutrophils (Fig. 6C, *P* < 0.01) and that SOD activity was elevated in SRA but not to a statistically significant level. We were not able to detect biologically active CAT using either a colorimetric or fluorometric enzyme activity assay (data not shown).

### 3.6 miRNA regulation of RA neutrophil gene expression

**Fig. 7.**
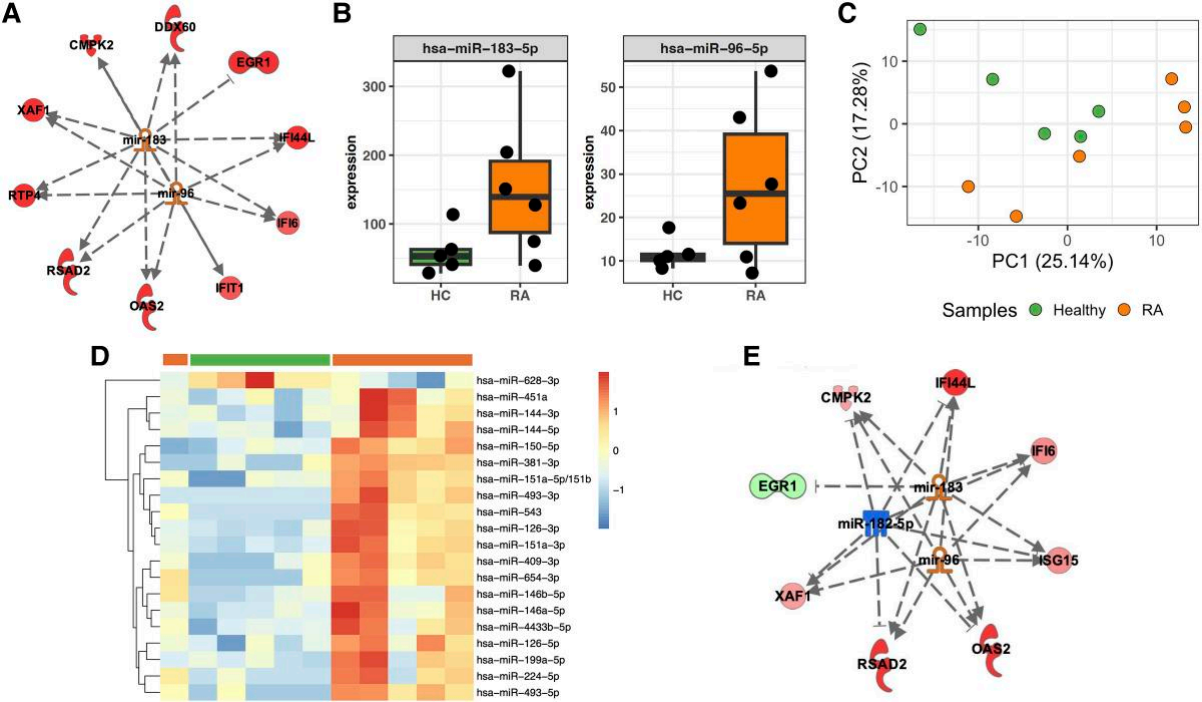
miRNA expression in SRA neutrophils. A) Predicted miRNA expression in SRA neutrophils from IPA analysis of transcriptomics data showing hub miRNAs miR-183 and miR-96 and expression of IFN-response genes (upregulated in red). B) Measured expression of miR-183-5p and miR-96-5p in paired SRA (orange) and HC (green) neutrophils (*P* < 0.05, adj. *P* > 0.05). C) PCA analysis of HC and SRA miRNAseq data. D) Heatmap of significantly expressed miRNAs from SRA (orange) and HC (green) neutrophils measured by miRNAseq (adj. *P* < 0.05). Color range is from logFC of −2 to +2, chosen because it is representative of the distribution of the expression data without being dominated by outliers. E) Network reconstruction using IPA of IFN-response genes and miRNAs expressed in RA neutrophils (red = upregulated mRNA, green = downregulated mRNA, orange = upregulated miRNA, blue = downregulated miRNA).

We next wanted to determine the effect of micro RNAs on neutrophil mRNA expression in SRA. IPA upstream regulator analysis predicted that the type-I IFN-response gene signature was regulated by miRNAs miR-183 (Fig. 7A, *P* = 4.30×10^−9^; Table S4) and miR-96 (Fig. 7A, *P* = 2.15×10^−8^; Table S4).^52^ We therefore performed miRNA sequencing on duplicate samples from a subset of SRA (*n* = 6) and HC (*n* = 5) neutrophils. We observed increased expression of miR-183 (Fig. 7B, *P* < 0.01, adj. *P* = 0.1) and miR-96 (Fig. 7B, *P* < 0.05, adj. *P* = 0.24) in SRA neutrophils; however, neither reached significance after *P*-value adjustment. There was clear separation of SRA and HC miRNAseq samples by principal component analysis (PCA, Fig. 7C), and we identified 20 miRNAs with significantly different expression in SRA neutrophils (Fig. 7D, adj. *P* < 0.05). Using the mRNA:miRNA filter function in IPA, we confirmed that IFN-α/beta signaling was the most highly predicted canonical signaling pathway activated in SRA neutrophils (adj. *P* = 1.17×10^−6^), with miRNAs miR-182-5p, miR-183, and miR-96 predicted to be regulating IFN-response gene expression (Fig. 7E, all *P* < 0.01; Table S5). The IFN-α receptor was predicted to be activated (*P* = 7.28×10^−14^) as were the IFN-activated transcription factors IRF1, IRF3, ORF7, and STAT1 (*P* < 0.01, Table S5). In addition, IPA predicted the activation of key regulators of mRNA and miRNA expression AGO2 (*P* = 1.97×10^−45^) and DICER1 (*P* = 3.33×10^−15^).

### 3.7 Identification of neutrophil subset gene expression signatures

**Fig. 8.**
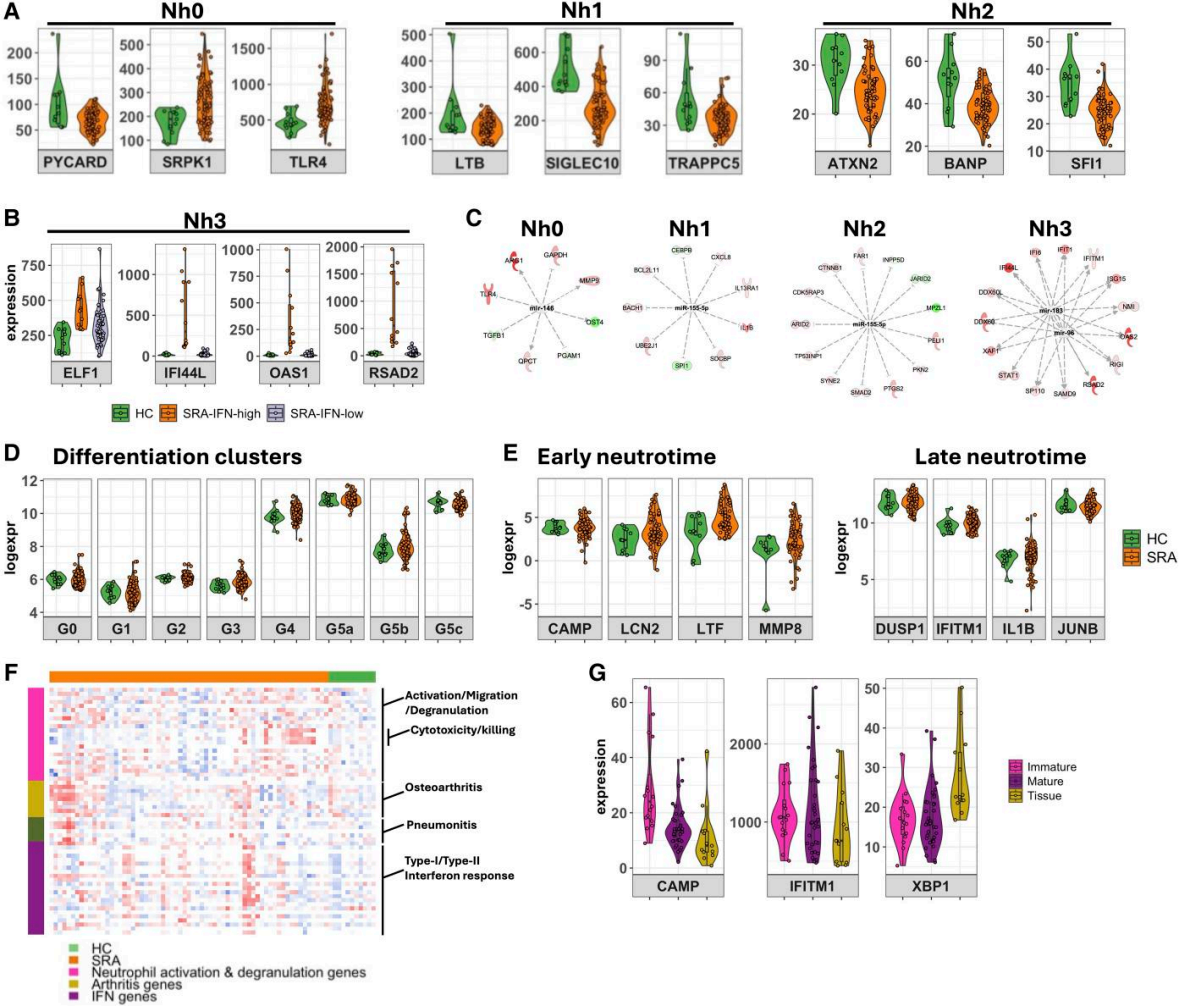
Identification of neutrophil subsets in SRA neutrophils using publicly available single-cell RNAseq datasets. A) Expression levels of gene markers of human neutrophil (Nh) subsets in SRA (orange) and HC (green) neutrophils (Nh0, Nh1, Nh2, and Nh3, adj. *P* < 0.05). B) Nh3 subset characterized by SRA IFN high and low patients. C) Predicted miRNA-regulated gene networks for each Nh subset. D) Expression of neutrophil differentiation cluster marker genes in SRA and HC neutrophils. E) Expression of early and late “neutrotime” genes in SRA and HC neutrophils. F) GSVA analysis of SRA (orange) and HC (green) neutrophils identifies gene signatures relating to early neutrophil activation (pink), IFN genes (purple), arthritis genes (yellow), and systemic inflammation associated with tissue inflammation (pneumonitis genes [dark green]). G) Expression levels of marker genes associated with polarization of early/immature neutrophils (*CAMP*, pink), mature neutrophils (*IFITM1*, purple), and tissue-specific inflammation (*XBP1*, yellow) across the SRA cohort.

Recent insight from single-cell RNA studies of mouse and human neutrophils has identified subsets of blood neutrophils, which can be defined by distinct expression of genes that are not necessarily expressed at the protein level. A study by Wigerblad et al.^53^ identified the trajectory of healthy human neutrophils from immature (Nh0) to intermediate (Nh1) stages, which then diverge to either Nh2 or Nh3 mature polarized neutrophils, based on the expression of distinct transcription factor genes. The Nh3 gene cluster, representing around 7% of blood neutrophils in healthy donors, includes a number of IFN-response genes including *IFI6*, *IFIT1*, and *RSAD2*. Using the published list of Nh marker genes,^53^ we compared the level of expression of genes representing each neutrophil subtype in our bulk RNAseq data from SRA and HC neutrophils. We found that 2 Nh0 genes, *SRPK1* and *TLR4*, were significantly higher in SRA neutrophils (Fig. 8A, adj. *P* < 0.05), whereas the Nh0 gene *PYCARD* was higher in HC (Fig. 8A, adj. *P* < 0.05). Three Nh1 marker genes, *LTB, SIGLEC10*, and *TRAPPC5*, were significantly lower in SRA neutrophils (Fig. 8A, adj. *P* < 0.05). Twenty-one genes marking the Nh2 cluster were significantly different in SRA neutrophils. Of these, 12 were lower in SRA neutrophils including ataxin-2, BTG3 associated with nuclear protein, and *SFI1* (Fig. 8A, adj. *P* < 0.05). One Nh3 gene, *ELF1*, was significantly elevated in SRA neutrophils, and the most upregulated genes based on fold change were the IFN-regulated genes *IFI44L, RSAD2*, and *OAS1* (adj. *P* < 0.1) due to the previously described IFN-high and IFN-low subpopulations of SRA patients (Fig. 7B). We used IPA to predict which micro RNAs were regulating the expression of SRA genes from each Nh cluster. Elevated expression of Nh0 genes was predicted to be regulated by the decrease in expression of miR-146a-5p (adj. *P* = 7.96×10^−3^), Nh1 and Nh2 genes were downregulated by increased expression of miR-155-5p (Nh1 adj. *P* = 1.10×10^−6^; Nh2 adj. *P* = 6.14×10^−5^), and Nh3 genes were predicted to be regulated by miR-183 (adj. *P* = 1.39×10^−22^) and miR-96 (adj. *P* = 4.86×10^−22^, Fig. 8C). Interestingly, the immature to mature Nh clusters from the Wigerblad et al. study^53^ closely mirrored a similar single-cell study in mouse neutrophils by Xie et al.^54^. In this study, neutrophil clusters G0 to G4 resembled immature, bone marrow neutrophils, with a mature G5 blood neutrophil population being subset into G5a (migratory/inflammatory genes), G5b (IFN-response genes), and G5c (aged/apoptotic neutrophils).^54^ While we did not see significantly different expression of any of the G-subset marker genes between SRA and HC neutrophils, clear subpopulations of SRA patients could be observed within the G0-G4 (immature), G5a (migratory/inflammatory), and G5b (IFN response) subsets (Fig. 8D).

In another study, Grieshaber-Bouyer et al.^55^ performed single-cell RNAseq analysis of mouse neutrophils, which identified subclusters of neutrophils with distinct gene expression profiles that track maturity along a trajectory from bone marrow into peripheral blood (termed “neutrotime”), with polarized states along neutrotime arising as a result of inflammatory stimulus and tissue-specific gene expression. Distinct neutrotime clusters were associated with gene expression patterns unique to both maturity state and the site of inflammation, which included KxB/N inflammatory arthritis model, IL-1β-induced pneumonitis, and IL-1β-induced peritonitis. We therefore wanted to determine whether we could detect different clusters of neutrotime gene signatures amongst our bulk RNAseq datasets. First, we compared the expression of early neutrotime genes (*CAMP, LCN2, LTF,* and *MMP8*) and late neutrotime genes (*DUSP1, IFITM1, IL1B,* and *JUNB*) in our SRA and HC transcriptomes (Fig. 8E). We found that while none of these genes had significantly different expression between the SRA and HC groups, a clear subset of SRA patients with high expression of early neutrotime genes was evident (Fig. 8E), suggesting that the population of immature neutrophils may be increased in these individuals.

We next performed GSVA of our SRA and HC neutrophil transcriptomes against the MSigDB including gene ontology sets and human phenotype signatures to investigate potential neutrotime signatures. This analysis allows to us to collapse our gene level information to relevant pathways or processes, in this case immune-based, while retaining sample-specific information. We found that the SRA transcriptomes had distinct phenotypes, relating to 1 of 4 molecular signatures: (i) neutrophil activation and degranulation, (ii) IFN-regulated genes, (iii) arthritis, and (iv) pneumonitis (Fig. 8F). These broadly relate to gene markers associated with neutrotime polarization states representing immature neutrophils (*CAMP*), mature neutrophils (*IFITM1*), and tissue-specific neutrophils (*XBP1*) (Fig. 8G). This finding once again highlights the increased presence of immature neutrophils in the circulation of people with SRA and not healthy participants.

## 4. Discussion

In this study, we have generated transcriptomic data from early and SRA and HC neutrophils to investigate the signaling pathways that contribute to neutrophil-driven inflammation in RA. Our analyses revealed a significant increase expression of genes of the type-I (IFN-α/β) signaling pathway in neutrophils from a subpopulation of people with early and severe DMARD-refractory RA. We identified a cluster of miRNAs predicted to regulate type-I IFN-response gene expression. We also identified expression of marker genes associated with subpopulations of blood neutrophils previously identified in single-cell RNAseq studies that are predicted to be regulated by specific miRNAs.^53–55^

The most profound signature that characterized RA neutrophils was a type-I IFN-induced signature. IFNs are a group of functionally related cytokines that are produced in response to viral infection.^56^ Human neutrophils are highly responsive to IFNs, which can alter the rate of apoptosis, enhance TNF-α-primed ROS production, and augment cytokine and chemokine synthesis.^25,57^ IFNs signal through dedicated IFN-α/β or IFN-γ receptors, which activate Janus Kinase (JAK)-STAT signaling and IRF family transcription factors, and the small molecule JAK inhibitors baricitinib and tofacitinib can modulate RA neutrophil activation.^57,58^ Our analysis showed that IFN-induced gene expression was the most significant signature in SRA neutrophils, and this activation signature was also evident in DMARD-naïve ERA neutrophils. Interestingly, our analysis suggests that gene expression in ERA and SRA neutrophils is remarkably similar, possibly reflecting uncontrolled, systemic inflammation. All RA patients in our study had active inflammation, with DAS28 scores >3.2 in the ERA cohort and >5.1 in the SRA cohort.

The IFN-response genes identified in SRA neutrophils play important roles in cellular inflammation and neutrophil activation. Oligoadenylate synthase (OAS) genes encode enzymes that sense exogenous nucleic acids and initiate antiviral pathways. High levels of OAS family genes are strongly associated with autoimmune diseases, including RA.^59–61^  *RSAD2* encodes an IFN-inducible antiviral protein of the S-adenosyl-L methionine enzyme superfamily that can regulate both immune cell proliferation and Th2 cell polarization via NF-κB, GATA3, and IL-4.^62^  *CMPK2* is a mitochondrial enzyme crucial for maintaining mitochondrial DNA integrity. It is located on the short arm of chromosome 2 (2p), positioned inverted and adjacent to the *RSAD2* gene.^63^ Both these genes are cotranscribed upon the induction of IFN-induced signaling.^64,65^ Altered levels of *CMPK2* have been reported to disrupt mitochondrial physiology and significantly deregulate macrophage homeostasis. Constitutive overexpression of the *CMPK2* in macrophages induces mitochondrial stress with marked depolarization of membrane potential, increases expression of proinflammatory genes (IL-8, IL-1β, and TNF-α), and enhances ROS production. Interestingly, sustained *CMPK2* expression results in a heightened glycolytic activity in macrophages^66^ something that is also observed in RA neutrophils.^58^  *IFI44L* typically modulates cellular environments to make them hostile to viruses, but dysregulation of this process may promote inflammation and targeting of self-tissues. High *IFI44L* levels correlate with RA disease activity,^67^ and may be a useful diagnostic tool to measure disease severity.^68^  *IFI16* is a sensor for exogenous DNA in macrophages, responsible for stimulating early innate immune responses and inflammation via the activation of inflammasomes. These cytoplasmic complexes are capable of processing and releasing proinflammatory IL-1β.^69^ Furthermore, *IFI16* activates stimulator of IFN genes (STING), consequently inducing NF-κB signaling in a cyclic guanosine monophosphate-AMP synthase (cGAS)-independent manner. As well as recognizing microbial DNA, cGAS can bind self-DNA leaked from mitochondria or the nucleus effectively triggering extensive inflammatory responses.^70^ Increased expression of cGAS-STING could therefore indicate a role for NETs in driving neutrophil activation and type-I IFN-response genes in RA.^71^ As well as detecting elevated NETs in SRA sera in this study, we recently identified a subpopulation of DESpR positive, NETting neutrophils in whole blood smears from people with RA^23^ confirming that NET production takes place in circulating RA blood as well as within synovial joints and tissue in RA.^6,9^ A recent clinical study also detected elevated NET debris (DNA:elastase and DNA:histone H3 complexes) in RA plasma, although this did not reach statistical significance or correlate with disease activity.^72^

Our bioinformatics analyses identified a key role of miRNAs miR-182-5p, miR-183, and miR-96 in regulating RA neutrophil gene expression. These miRNAs exist in a cluster known as miR-183C.^73^ This cluster is highly conserved and is transcribed as a polycistronic miRNA cluster, known to promote cancer development. These miRNAs possess almost identical seed sequences, and this similarity enables them to act cooperatively.^74^ The miR-183C cluster positively regulates type-1 IFN production by inhibiting negative regulators of the antiviral response. Specifically, miR-183C targets and silences PP2A, a known inhibitor of transcription factors STAT1 and IRF3^75,76^ leading to enhanced IRF3 signaling in the initiation of type-1 IFN production.^69^ The miR-183C cluster is also known to play roles in immunity and autoimmunity, with its dysregulation reported in various autoimmune disorders including multiple sclerosis, systemic lupus erythematosus (SLE), and ocular autoimmune disorders.^77^ In murine models of SLE and multiple sclerosis, inhibition of this cluster in vivo has yielded positive therapeutic outcomes, with a significant attenuation of disease symptoms.^77–79^ This has led to the prospect of developing miR-183C miRNA-based therapies for targeting autoimmune diseases.

By comparing our bulk RNAseq data with single-cell RNAseq datasets, we were able to identify gene signatures relating to neutrophil subsets. Three recent publications have shed new insight onto the complexity and heterogeneity of the blood neutrophil population. A single-cell RNAseq study of mouse neutrophils identified a transcriptional trajectory program during neutrophil development, maturation, and migration into tissues that was termed “neutrotime.”^55^ Each maturation step could be identified by marker genes, and indeed we detected these gene signatures in our SRA neutrophils. We found that we could identify a clear subset of patients who had neutrophils expressing either early neutrotime or late neutrotime marker genes. The early neutrotime genes code for neutrophil granule proteins such as LTF and lipocalin-2, which we have previously reported to be highly expressed in a subset of SRA patients and in SRA low-density granulocytes.^14,51^ These genes are normally silenced during neutrophil development before mature neutrophils exit the bone marrow and enter circulation,^80^ and this could indicate high levels of inflammation and increased granulopoiesis in these individuals. An additional mouse neutrophil single-cell study^54^ identified 4 subsets of immature neutrophils within the bone marrow compartment and 3 mature neutrophil subsets within blood with distinct gene expression profiles relating to specific neutrophil functions. A clear subset of SRA patients exhibited the G5b subset gene expression profile, which relates to expression of IFN-response genes. In addition, a handful of patients expressed genes associated with immature bone marrow neutrophils (G0 to G4) once again underlying the presence of immature neutrophils within whole neutrophil blood populations. We were also able to compare our bulk RNAseq data with a human neutrophil single-cell RNAseq study,^53^ which again identified distinct maturation subsets of blood neutrophils from healthy individuals. Our analysis showed that SRA neutrophils expressed genes for all Nh subsets identified in this study, and in addition, we found a subset of SRA patients with increased expression of Nh3 IFN marker genes. We did not identify elevated serum levels of IFN-α in the patients with high expression of IFN-response genes raising the question as to whether it is indeed IFNs that are activating IFN-response genes in SRA or whether it is some other agonist such as viral or NET-derived DNA via the cGAS-STING system. We also predicted regulation of neutrophil Nh subsets by miRNAs miR-146a-5p and miR-155-5p. Decreased expression of miR-146a-5p has been shown to increase expression of genes such as *TRAF6* and *IRAK1*, which are involved in activating NF-κB signaling and promoting inflammation,^81^ whereas in mice, miR-155 has been identified as a key regulator of PAD4 activation and NET release.^82^

As well as altered gene expression, we identified an altered, activated phenotype in our SRA neutrophil cohort. Our gene overrepresentation analysis identified several ontologies relating to the response to oxidative stress, hydrogen peroxide, and ROS in SRA neutrophils. Enzyme activity assays revealed higher levels of SOD activity in some, but not all, RA patients compared with healthy controls. This enzyme is involved in the reduction of the superoxide radical to hydrogen peroxide eg during the neutrophil respiratory burst.^11^ In contrast, we detected significantly lower levels of GPx activity in RA neutrophils. This enzyme plays an important role in detoxification of hydrogen peroxide by reducing it to water. Taken together, this evidence would suggest that RA neutrophils have a dysregulation of hydrogen peroxide detoxification, which would cause oxidative stress leading to the production of hypochlorous acid through the action of myeloperoxidase. As well as inducing damage to tissues, DNA, and protein, low levels of hypochlorous acid can rapidly deactivate GPx^83^ suggesting a possible intracellular mechanism for the results observed.

We recognized several limitations to our study. Our blood samples were nonfasted and collected from multiple clinics at different times of day. While there is mounting evidence of the effect of metabolism and circadian rhythms on neutrophil activation and response to infection,^58,84^ we did not identify any gene expression patterns relating to the time of blood collection in our data (data not shown). In addition, we measured RNA expression in blood neutrophils isolated by density centrifugation, meaning we likely lost low-density neutrophils and, potentially, fragile NETting neutrophils during the isolation process.^23^ We were also unable to obtain neutrophils from the synovial joint as part of this study, although we have previously reported the distinct gene expression profile of RA synovial fluid neutrophils.^9^ Importantly, while the data we present here suggest an important role for micro RNAs in regulating neutrophil gene expression, we did not perform experiments to directly identify a causal link. As human neutrophils are terminally differentiated cells and difficult to transfect or manipulate in vitro, a causal link between specific miRNAs and mRNA expression could be established in the future by direct targeting of miRNAs in tractable models of neutrophil-like cells such as differentiated HL60 cells and/or HoxB8 cells. Despite these limitations, we believe our study sheds important new insight onto the complexity and heterogeneity of RA blood neutrophil gene expression and provides a potential mechanism for regulation of neutrophil subsets, and the IFN-response gene signature detected in a subset of people with RA, by miRNAs.

## 5. Conclusion

In conclusion, we have described a complex and heterogeneous neutrophil transcriptome across people with RA. These gene expression profiles are conserved across patients with ERA and severe, DMARD-refractory RA. The major gene signature within the cohort as a whole is an IFN-response gene signature, and we propose this is regulated by the miRNA hub of miR-182, miR-183, and miR-96 (miR-183C cluster). IFN-α was not significantly elevated in RA sera, but we have detected increased levels of circulating NET debris, which may activate IFN gene expression via the DNA-sensing genes (cGAS-STING). The heterogeneous transcriptomes measured over the population of people with RA can be broadly compared with single RNAseq datasets, which identify hub genes regulating polarized states of neutrophil subsets. These neutrophil subsets are predicted to be regulated by different miRNAs including miR-146a-5p, miR-155-5p, and the miR-183C cluster. We believe our study provides valuable insight into the activation of neutrophils in the blood of people with RA and defines the heterogeneity of neutrophil subsets in people with RA warranting further investigation by single-cell sequencing of RA blood neutrophil populations.

## Supplementary Material

qiaf090_Supplementary_Data

## Data Availability

The raw RNA sequencing data reported in this manuscript have been deposited in the NCBI Gene Expression Omnibus (GEO) and are accessible through GEO Series accession numbers GSE274598 (https://www.ncbi.nlm.nih.gov/geo/query/acc.cgi?acc=GSE274598) and GSE274996 (https://www.ncbi.nlm.nih.gov/geo/query/acc.cgi?acc=GSE274996). The processed microRNA data reported in this manuscript can be accessed via FigShare at: 10.6084/m9.figshare.27136062.
