# Supplementary material for: Complexity of the neutrophil transcriptome in early and severe rheumatoid arthritis: a role for microRNAs?
Source: J Leukoc Biol. 2025 Jun 18;117(6):qiaf090. doi: 10.1093/jleuko/qiaf090 (PMC12210129; doi:10.1093/jleuko/qiaf090)
Supplement: qiaf090_Supplementary_Data [file qiaf090_supplementary_data.docx]

**Supplementary Data**

**Complexity of the neutrophil transcriptome in early and severe rheumatoid arthritis. A role for microRNAs?**

Michele Fresneda Alarcon^1†^, Genna Ali Abdullah^1†^, John Alexander Beggs^2^, Isobel Kynoch^2^, Andrew Sellin^1^, Andrew Cross^1^, Sam Haldenby^3^, Philipp Antczak^4,5^, Eva Caamaño Gutiérrez^4,6^, Helen Louise Wright^1^*

^1^Institute of Life Course and Medical Sciences, University of Liverpool, Liverpool UK

^2^School of Biosciences, University of Liverpool, Liverpool UK

^3^Centre for Genomic Research, University of Liverpool, Liverpool UK

^4^ Computational Biology Facility, University of Liverpool, Liverpool UK

^5^ Centre for Molecular Medicine, University of Cologne, Cologne, Germany

^6^ Institute of Systems, Molecular and Integrative Biology, University of Liverpool UK

**Supplementary Table 1 – Upstream regulator analysis results for SRA vs HC mRNA.** Summary of predicted upstream cytokines and transcription regulators shown. Full upstream regulator analysis can be found here: 10.6084/m9.figshare.27968076

| **Upstream Regulator** | **Molecule Type** | **Predicted Activation State** | **Activation z-score** | **B-H corrected p-value** |
| --- | --- | --- | --- | --- |
| TNF | cytokine | Activated | 7.24 | 1.96E-36 |
| IFNG | cytokine | Activated | 8.739 | 2.58E-36 |
| STAT1 | transcription regulator | Activated | 6.055 | 5.89E-34 |
| STAT3 | transcription regulator | Activated | 2.38 | 5.89E-34 |
| IL4 | cytokine | Activated | 2.168 | 2.33E-33 |
| IL1B | cytokine | Activated | 7.033 | 4.00E-29 |
| NONO | transcription regulator | Activated | 6.22 | 7.86E-29 |
| IL2 | cytokine | Activated | 4.974 | 4.16E-27 |
| IFNA2 | cytokine | Activated | 5.966 | 1.98E-26 |
| CSF1 | cytokine | Activated | 3.611 | 2.65E-26 |
| ETV3 | transcription regulator | Inhibited | -5.385 | 2.95E-26 |
| IFNL1 | cytokine | Activated | 5.634 | 1.72E-23 |
| ETV6 | transcription regulator | Inhibited | -4.522 | 1.59E-20 |
| PRL | cytokine | Activated | 5.621 | 1.85E-20 |
| IL6 | cytokine | Activated | 3.522 | 2.09E-17 |
| IRF3 | transcription regulator | Activated | 5.109 | 9.69E-17 |
| CSF2 | cytokine | Activated | 4.338 | 1.07E-16 |
| SPI1 | transcription regulator | Activated | 2.983 | 8.76E-16 |
| IL15 | cytokine | Activated | 4.001 | 1.54E-15 |
| IRF7 | transcription regulator | Activated | 5.859 | 6.77E-15 |
| IL21 | cytokine | Activated | 3.751 | 1.21E-14 |
| IKZF1 | transcription regulator | Inhibited | -3.374 | 2.54E-14 |
| NFAT5 | transcription regulator | Inhibited | -2.638 | 2.93E-14 |
| EP300 | transcription regulator | Activated | 3.397 | 7.33E-14 |
| TP53 | transcription regulator | Activated | 2.279 | 7.57E-14 |
| IL27 | cytokine | Activated | 5.91 | 1.24E-13 |
| CD40LG | cytokine | Activated | 3.887 | 1.43E-13 |
| GATA2 | transcription regulator | Inhibited | -2.442 | 3.62E-13 |
| OSM | cytokine | Activated | 3.798 | 8.86E-13 |
| ZNF750 | transcription regulator | Inhibited | -4.737 | 8.86E-13 |
| PRDM1 | transcription regulator | Inhibited | -2.28 | 1.17E-12 |
| SP1 | transcription regulator | Activated | 3.44 | 1.42E-12 |
| CSF3 | cytokine | Activated | 2.843 | 2.35E-12 |
| IRF1 | transcription regulator | Activated | 4.572 | 4.40E-12 |
| IL18 | cytokine | Activated | 4.443 | 4.80E-12 |
| CEBPA | transcription regulator | Activated | 3.018 | 1.29E-11 |
| IFNA1/IFNA13 | cytokine | Activated | 3.893 | 1.62E-11 |
| NFKB1 | transcription regulator | Activated | 3.651 | 2.32E-11 |
| RELA | transcription regulator | Activated | 3.654 | 3.07E-11 |
| NUPR1 | transcription regulator | Activated | 2.188 | 4.27E-11 |
| IL1RN | cytokine | Inhibited | -3.946 | 5.88E-11 |
| FOXC1 | transcription regulator | Activated | 3.651 | 6.17E-11 |
| IL3 | cytokine | Activated | 2.625 | 8.55E-11 |
| TP63 | transcription regulator | Activated | 2.026 | 1.38E-10 |
| NFKBIA | transcription regulator | Activated | 2.292 | 1.71E-10 |
| IL1A | cytokine | Activated | 5.241 | 3.91E-10 |
| IFNB1 | cytokine | Activated | 3.774 | 5.02E-10 |
| CEBPB | transcription regulator | Activated | 3.413 | 7.49E-10 |
| SMARCA4 | transcription regulator | Activated | 2.7 | 7.74E-10 |
| HMG20A | transcription regulator | Activated | 4.379 | 9.19E-10 |
| JUN | transcription regulator | Activated | 3.292 | 1.71E-09 |
| IL17A | cytokine | Activated | 3.775 | 2.19E-09 |
| IL7 | cytokine | Activated | 3.793 | 2.28E-09 |
| TWIST1 | transcription regulator | Activated | 3.366 | 2.29E-09 |
| CBX5 | transcription regulator | Inhibited | -2.429 | 3.10E-09 |
| IFNL4 | cytokine | Activated | 2.956 | 3.27E-09 |
| IRF9 | transcription regulator | Activated | 3.356 | 9.59E-09 |
| HIF1A | transcription regulator | Activated | 4.223 | 2.16E-08 |
| NKX2-3 | transcription regulator | Inhibited | -3.942 | 2.49E-08 |
| GFI1 | transcription regulator | Inhibited | -2.654 | 2.60E-08 |
| STAT2 | transcription regulator | Activated | 3.051 | 3.12E-08 |
| MYBL2 | transcription regulator | Activated | 2.848 | 4.86E-08 |
| CTNNB1 | transcription regulator | Activated | 2.807 | 8.31E-08 |
| KLF6 | transcription regulator | Activated | 3.634 | 9.31E-08 |
| PML | transcription regulator | Activated | 2.646 | 9.52E-08 |
| NPM1 | transcription regulator | Activated | 4.056 | 1.69E-07 |
| MSC | transcription regulator | Activated | 3.207 | 2.30E-07 |
| EBF4 | transcription regulator | Activated | 2.623 | 2.80E-07 |
| THPO | cytokine | Activated | 2.081 | 3.27E-07 |
| TBX3 | transcription regulator | Activated | 3.2 | 3.43E-07 |
| BHLHE40 | transcription regulator | Activated | 4.91 | 3.80E-07 |
| CITED2 | transcription regulator | Inhibited | -4.48 | 5.38E-07 |
| CCL2 | cytokine | Activated | 2.819 | 6.80E-07 |
| IFNA4 | cytokine | Activated | 3.485 | 7.00E-07 |
| IFNE | cytokine | Activated | 3.302 | 7.88E-07 |
| NFATC2 | transcription regulator | Activated | 2.16 | 8.34E-07 |
| EPAS1 | transcription regulator | Activated | 3.745 | 8.34E-07 |
| IL5 | cytokine | Activated | 3.437 | 1.06E-06 |
| REL | transcription regulator | Activated | 2.269 | 1.12E-06 |
| BCL6 | transcription regulator | Inhibited | -2.968 | 2.64E-06 |
| CBFB | transcription regulator | Inhibited | -2.168 | 3.51E-06 |
| NOTCH1 | transcription regulator | Activated | 2.221 | 3.88E-06 |
| ETV7 | transcription regulator | Inhibited | -2.213 | 3.97E-06 |
| SMARCB1 | transcription regulator | Activated | 3.096 | 6.73E-06 |
| POU5F1 | transcription regulator | Activated | 2.964 | 8.89E-06 |
| ETS1 | transcription regulator | Activated | 2.528 | 9.00E-06 |
| FOXL2 | transcription regulator | Activated | 2.314 | 1.12E-05 |
| IKZF2 | transcription regulator | Inhibited | -3.162 | 1.14E-05 |
| NOTCH3 | transcription regulator | Activated | 3.514 | 1.48E-05 |
| WNT3A | cytokine | Activated | 3.666 | 1.98E-05 |
| EDN1 | cytokine | Activated | 3.596 | 2.19E-05 |
| EGR2 | transcription regulator | Activated | 2.086 | 2.39E-05 |
| IKZF3 | transcription regulator | Inhibited | -2.685 | 3.21E-05 |
| KLF7 | transcription regulator | Activated | 2.445 | 3.29E-05 |
| C5 | cytokine | Activated | 3.784 | 3.71E-05 |
| IFI16 | transcription regulator | Activated | 2.137 | 5.66E-05 |
| RB1 | transcription regulator | Inhibited | -2.312 | 5.87E-05 |
| TNFSF11 | cytokine | Activated | 2.738 | 5.97E-05 |
| STAT4 | transcription regulator | Activated | 4.051 | 7.24E-05 |
| RBPJ | transcription regulator | Activated | 2.431 | 8.36E-05 |
| HMGB1 | transcription regulator | Activated | 3.327 | 9.61E-05 |
| EGR1 | transcription regulator | Activated | 2.056 | 1.02E-04 |
| MITF | transcription regulator | Activated | 2.621 | 1.06E-04 |
| MED1 | transcription regulator | Activated | 2.366 | 1.14E-04 |
| SIRT1 | transcription regulator | Inhibited | -2.748 | 1.52E-04 |
| NFATC1 | transcription regulator | Activated | 2.577 | 1.57E-04 |
| JUNB | transcription regulator | Activated | 2.714 | 1.61E-04 |
| IFNK | cytokine | Activated | 2.449 | 1.77E-04 |
| IL36G | cytokine | Activated | 3.812 | 2.17E-04 |
| E2F1 | transcription regulator | Activated | 2.095 | 2.60E-04 |
| FOXM1 | transcription regulator | Activated | 2.121 | 2.69E-04 |
| CCND1 | transcription regulator | Activated | 2.328 | 2.86E-04 |
| CXCL12 | cytokine | Activated | 3.067 | 2.96E-04 |
| IRF5 | transcription regulator | Activated | 3.248 | 3.40E-04 |
| NCOA3 | transcription regulator | Activated | 2.376 | 3.70E-04 |
| ATF4 | transcription regulator | Activated | 2.685 | 4.19E-04 |
| PPRC1 | transcription regulator | Activated | 2.949 | 4.51E-04 |
| ELF4 | transcription regulator | Activated | 2.576 | 4.52E-04 |
| ZFP36 | transcription regulator | Inhibited | -3.427 | 4.83E-04 |
| CCL11 | cytokine | Activated | 2.918 | 5.15E-04 |
| YBX1 | transcription regulator | Activated | 3.446 | 5.43E-04 |
| TEAD4 | transcription regulator | Activated | 2.92 | 5.52E-04 |
| POU2F2 | transcription regulator | Activated | 2.891 | 5.86E-04 |
| TIMP1 | cytokine | Activated | 2.376 | 7.67E-04 |
| TNFSF14 | cytokine | Activated | 2.414 | 8.62E-04 |
| IFNA14 | cytokine | Activated | 2.197 | 9.77E-04 |
| IFNA10 | cytokine | Activated | 2.197 | 9.77E-04 |
| IFNA21 | cytokine | Activated | 2.193 | 9.77E-04 |
| IFNA5 | cytokine | Activated | 2.197 | 9.77E-04 |
| IFNA7 | cytokine | Activated | 2.197 | 9.77E-04 |
| IFNA6 | cytokine | Activated | 2.197 | 9.77E-04 |
| CCL20 | cytokine | Activated | 2.158 | 1.16E-03 |
| BCOR | transcription regulator | Inhibited | -2 | 1.29E-03 |
| HIF3A | transcription regulator | Inhibited | -2.2 | 1.33E-03 |
| IFNA8 | cytokine | Activated | 2.214 | 1.33E-03 |
| IFNA16 | cytokine | Activated | 2.197 | 1.33E-03 |
| CREB1 | transcription regulator | Activated | 2.97 | 1.39E-03 |
| HIVEP1 | transcription regulator | Inhibited | -2.343 | 1.45E-03 |
| CCL5 | cytokine | Activated | 2.339 | 1.69E-03 |
| ZBTB10 | transcription regulator | Activated | 3.035 | 2.08E-03 |
| ELK1 | transcription regulator | Activated | 2.771 | 2.20E-03 |
| IL22 | cytokine | Activated | 2.696 | 2.43E-03 |
| TRIM24 | transcription regulator | Inhibited | -3.592 | 2.43E-03 |
| KDM3A | transcription regulator | Activated | 2.19 | 2.53E-03 |
| FOXA2 | transcription regulator | Activated | 2.77 | 2.67E-03 |
| MAML1 | transcription regulator | Activated | 2.236 | 2.85E-03 |
| EHF | transcription regulator | Activated | 2.891 | 2.93E-03 |
| CRH | cytokine | Activated | 2.412 | 2.98E-03 |
| WBP2 | transcription regulator | Activated | 2.138 | 3.67E-03 |
| TNFSF12 | cytokine | Activated | 2.23 | 3.91E-03 |
| IL32 | cytokine | Activated | 2.606 | 4.07E-03 |
| NFYA | transcription regulator | Activated | 2.433 | 4.50E-03 |
| SOX3 | transcription regulator | Inhibited | -2.53 | 4.60E-03 |
| CXCL8 | cytokine | Activated | 2.919 | 4.67E-03 |
| POU2AF1 | transcription regulator | Activated | 2.356 | 5.66E-03 |
| GMNN | transcription regulator | Inhibited | -2.333 | 5.89E-03 |
| NCOR1 | transcription regulator | Inhibited | -2.137 | 8.29E-03 |
| TCF7L2 | transcription regulator | Activated | 3.645 | 8.30E-03 |
| IL12B | cytokine | Activated | 2.596 | 8.81E-03 |
| SOX1 | transcription regulator | Inhibited | -2.333 | 8.81E-03 |
| SPP1 | cytokine | Activated | 2.737 | 8.92E-03 |
| NFKB2 | transcription regulator | Activated | 3.207 | 1.11E-02 |
| USF1 | transcription regulator | Activated | 2.412 | 1.24E-02 |
| PRDM16 | transcription regulator | Inhibited | -2.138 | 1.34E-02 |
| JARID2 | transcription regulator | Activated | 2.423 | 1.42E-02 |
| TEAD3 | transcription regulator | Activated | 2.63 | 1.55E-02 |
| IRF2BP2 | transcription regulator | Inhibited | -2.716 | 1.76E-02 |
| HOXB4 | transcription regulator | Activated | 2 | 1.76E-02 |
| PLAG1 | transcription regulator | Activated | 2.449 | 2.44E-02 |
| NOSTRIN | transcription regulator | Inhibited | -2.216 | 2.49E-02 |
| LIF | cytokine | Activated | 2.021 | 2.62E-02 |
| EBI3 | cytokine | Activated | 2.021 | 2.80E-02 |
| SREBF1 | transcription regulator | Activated | 2.302 | 3.02E-02 |
| RBL1 | transcription regulator | Inhibited | -2.021 | 3.07E-02 |
| TNFSF15 | cytokine | Activated | 2.574 | 3.17E-02 |
| WT1 | transcription regulator | Activated | 2.067 | 3.22E-02 |
| ARNT2 | transcription regulator | Activated | 3.357 | 3.62E-02 |
| TEAD2 | transcription regulator | Activated | 2.449 | 3.98E-02 |
| SIX5 | transcription regulator | Inhibited | -2 | 4.41E-02 |

**Supplementary Table 2 – Upstream regulator analysis results for ERA vs HC mRNA.** Summary of predicted upstream cytokines and transcription regulators shown. Full upstream regulator analysis can be found here: 10.6084/m9.figshare.27968157

| **Upstream Regulator** | **Molecule Type** | **Predicted Activation State** | **Activation z-score** | **B-H corrected p-value** |
| --- | --- | --- | --- | --- |
| IFNG | cytokine | Activated | 7.106 | 2.71E-34 |
| STAT1 | transcription regulator | Activated | 5.519 | 1.30E-31 |
| ETV3 | transcription regulator | Inhibited | -5.385 | 5.97E-29 |
| NONO | transcription regulator | Activated | 6.381 | 1.68E-28 |
| CSF1 | cytokine | Activated | 3.008 | 8.31E-25 |
| IFNL1 | cytokine | Activated | 5.63 | 1.19E-23 |
| TNF | cytokine | Activated | 5.811 | 1.13E-22 |
| ETV6 | transcription regulator | Inhibited | -4.882 | 9.43E-22 |
| PRL | cytokine | Activated | 5.907 | 1.35E-19 |
| ZNF750 | transcription regulator | Inhibited | -4.879 | 4.27E-19 |
| IRF7 | transcription regulator | Activated | 6.168 | 7.66E-19 |
| IL1B | cytokine | Activated | 5.657 | 8.57E-19 |
| IRF1 | transcription regulator | Activated | 4.845 | 2.16E-16 |
| IFNA2 | cytokine | Activated | 5.745 | 5.42E-15 |
| IL27 | cytokine | Activated | 4.855 | 1.33E-13 |
| IRF3 | transcription regulator | Activated | 5.214 | 1.45E-13 |
| OSM | cytokine | Activated | 4.221 | 1.10E-12 |
| FOXC1 | transcription regulator | Activated | 3.543 | 3.93E-11 |
| IL6 | cytokine | Activated | 3.504 | 4.96E-11 |
| IL1RN | cytokine | Inhibited | -3.19 | 4.96E-11 |
| SPI1 | transcription regulator | Activated | 3.88 | 2.23E-10 |
| IRF9 | transcription regulator | Activated | 3.514 | 3.84E-10 |
| CEBPA | transcription regulator | Activated | 2.405 | 5.37E-10 |
| TWIST1 | transcription regulator | Activated | 2.866 | 1.32E-09 |
| CTNNB1 | transcription regulator | Activated | 2.147 | 1.37E-09 |
| STAT2 | transcription regulator | Activated | 2.449 | 1.44E-09 |
| MSC | transcription regulator | Activated | 3 | 1.44E-09 |
| IFNL4 | cytokine | Activated | 2.956 | 2.24E-09 |
| IFNA1/IFNA13 | cytokine | Activated | 3.904 | 4.77E-09 |
| IFNB1 | cytokine | Activated | 3.962 | 5.86E-09 |
| HMG20A | transcription regulator | Activated | 3.714 | 8.24E-09 |
| PRDM1 | transcription regulator | Inhibited | -3.775 | 1.83E-08 |
| PML | transcription regulator | Activated | 2.69 | 2.14E-08 |
| CD40LG | cytokine | Activated | 2.261 | 5.19E-08 |
| NKX2-3 | transcription regulator | Inhibited | -3.788 | 6.32E-08 |
| EP300 | transcription regulator | Activated | 2.571 | 6.56E-08 |
| IKZF1 | transcription regulator | Inhibited | -3.447 | 9.06E-08 |
| CSF3 | cytokine | Activated | 2.653 | 1.18E-07 |
| IL1A | cytokine | Activated | 3.294 | 1.82E-07 |
| IL17A | cytokine | Activated | 2.666 | 1.97E-07 |
| SP1 | transcription regulator | Activated | 3.008 | 3.41E-07 |
| CBX5 | transcription regulator | Inhibited | -2.357 | 4.75E-07 |
| IL2 | cytokine | Activated | 2.247 | 9.57E-07 |
| JUNB | transcription regulator | Activated | 3.426 | 1.17E-06 |
| RELA | transcription regulator | Activated | 2.483 | 2.35E-06 |
| BHLHE40 | transcription regulator | Activated | 3.743 | 3.92E-06 |
| NOTCH3 | transcription regulator | Activated | 2.554 | 1.78E-05 |
| CITED2 | transcription regulator | Inhibited | -2.738 | 4.73E-05 |
| KLF6 | transcription regulator | Activated | 3.004 | 4.73E-05 |
| SMARCB1 | transcription regulator | Activated | 2.947 | 5.34E-05 |
| THPO | cytokine | Activated | 2.091 | 5.72E-05 |
| WNT3A | cytokine | Activated | 2.316 | 6.00E-05 |
| IKZF3 | transcription regulator | Inhibited | -3.45 | 8.89E-05 |
| NPM1 | transcription regulator | Activated | 2.53 | 1.17E-04 |
| MYBL2 | transcription regulator | Activated | 2.136 | 2.00E-04 |
| KLF7 | transcription regulator | Activated | 2.231 | 3.25E-04 |
| TRIM24 | transcription regulator | Inhibited | -3.526 | 4.61E-04 |
| EPAS1 | transcription regulator | Activated | 2.335 | 5.25E-04 |
| NFATC2 | transcription regulator | Activated | 2.14 | 6.20E-04 |
| IFNA4 | cytokine | Activated | 2.889 | 6.53E-04 |
| WBP2 | transcription regulator | Activated | 2.324 | 7.75E-04 |
| IFNE | cytokine | Activated | 2.646 | 1.08E-03 |
| IFNK | cytokine | Activated | 2.236 | 1.20E-03 |
| PPARGC1A | transcription regulator | Inhibited | -2.251 | 1.24E-03 |
| IRF4 | transcription regulator | Inhibited | -2.982 | 1.29E-03 |
| IRF5 | transcription regulator | Activated | 3.095 | 2.63E-03 |
| IL33 | cytokine | Activated | 2.226 | 2.65E-03 |
| IL18 | cytokine | Activated | 3.016 | 2.78E-03 |
| PRDM16 | transcription regulator | Inhibited | -2.375 | 3.56E-03 |
| CCL5 | cytokine | Activated | 2.159 | 4.18E-03 |
| IL36G | cytokine | Activated | 2.884 | 4.91E-03 |
| SIRT1 | transcription regulator | Inhibited | -2.207 | 8.02E-03 |
| ZBTB10 | transcription regulator | Activated | 3.464 | 8.06E-03 |
| IFI16 | transcription regulator | Activated | 2.359 | 8.51E-03 |
| IL21 | cytokine | Activated | 2.704 | 1.00E-02 |
| YY1 | transcription regulator | Activated | 2.003 | 1.06E-02 |
| SNAI2 | transcription regulator | Activated | 2.274 | 1.06E-02 |
| TIMP1 | cytokine | Activated | 2.621 | 1.08E-02 |
| HIF1A | transcription regulator | Activated | 2.949 | 1.11E-02 |
| ID2 | transcription regulator | Inhibited | -2.043 | 1.26E-02 |
| IL7 | cytokine | Activated | 2.728 | 1.29E-02 |
| C5 | cytokine | Activated | 2.953 | 1.44E-02 |
| GATA1 | transcription regulator | Activated | 2.145 | 1.58E-02 |
| TCF7L2 | transcription regulator | Activated | 3.555 | 1.82E-02 |
| PPRC1 | transcription regulator | Activated | 2.401 | 2.55E-02 |
| CCL11 | cytokine | Activated | 2.378 | 2.78E-02 |
| NFKB2 | transcription regulator | Activated | 2.536 | 3.34E-02 |
| HMGB1 | transcription regulator | Activated | 2.594 | 3.47E-02 |
| ESX1 | transcription regulator | Inhibited | -2.449 | 4.06E-02 |
| IL24 | cytokine | Activated | 2.385 | 4.70E-02 |
| FOXA2 | transcription regulator | Activated | 2.026 | 4.86E-02 |

**Supplementary Table 3 – ARACNE2 gene expression network modules.** Gene ontology over-representation analysis was performed using BINGO (GO) and canonical pathway enrichment was performed using IPA.

| **Module** | **Method used** | **Adjusted**  **p-value** |
| --- | --- | --- |
| **M1. Metabolism & Transcription**  **(880 nodes, 19,281 edges)** |  |  |
| Regulation of gene expression | GO | 0.00018665 |
| Nucleobase, nucleoside, nucleotide and nucleic acid metabolic process | GO | 0.00018665 |
| Cellular metabolic process | GO | 0.0027225 |
| Valine degradation I | IPA | 0.00295121 |
| EIF2 signaling | IPA | 0.00512861 |
| mRNA processing | GO | 0.014741 |
| RNA transport | GO | 0.017427 |
| Cellular response to stress | GO | 0.024697 |
| Establishment of protein localization | GO | 0.034844 |
| Protein modification by small protein conjugation or removal | GO | 0.039013 |
|  |  |  |
| **M2. Integrin & Cytokine Receptors**  **(786 nodes, 25,308 edges)** |  |  |
| Intracellular signal transduction | GO | 7.0754E-08 |
| Production of NO and ROS in Macrophages | IPA | 1.122E-07 |
| NFAT in regulation of the immune response | IPA | 3.7154E-06 |
| Integrin signaling | IPA | 4.0738E-06 |
| IL-8 Signaling | IPA | 4.4668E-06 |
| IL-6 Signaling | IPA | 5.2481E-06 |
| Signaling by Rho family GTPases | IPA | 9.7724E-06 |
| Post-translational protein modification | GO | 1.1237E-05 |
| HMGB1 signaling | IPA | 1.3183E-05 |
| NF-kappaB signaling | IPA | 3.9811E-05 |
|  |  |  |
| **M3. Kinase Signalling**  **(394 nodes, 1,855 edges)** |  |  |
| Cellular macromolecule metabolic process | GO | 2.1E-15 |
| Protein modification process | GO | 4.73E-09 |
| AMPK signaling | IPA | 8.3176E-07 |
| Establishment of protein localization | GO | 1.18E-06 |
| Vesicle-mediated transport | GO | 1.94E-06 |
| Cdc42 signaling | IPA | 6.3096E-06 |
| NF-kappaB signaling | IPA | 0.00026915 |
| CD40 signaling | IPA | 0.00085114 |
| 3-phosphoinositide biosynthesis | IPA | 0.00218776 |
| SAPK/JNK signaling | IPA | 0.00186209 |
|  |  |  |
| **M4. Interferon & Toll-like Receptors**  **(89 nodes, 295 edges)** |  |  |
| Interferon signaling | IPA | 3.9811E-23 |
| Response to virus | GO | 3.0548E-15 |
| Activation of IRF by cytosolic pattern recognition receptors | IPA | 5.0119E-14 |
| Immune response | GO | 4.3147E-07 |
| Retinoic acid mediated apoptosis signaling | IPA | 3.6308E-06 |
| Role of RIG1-like receptors in antiviral innate immunity | IPA | 2.5704E-05 |
| Death receptor signaling | IPA | 0.00044668 |
| Innate immune response | GO | 0.0011464 |
| Regulation of defense response | GO | 0.0080641 |
| Regulation of defense response to virus | GO | 0.090327 |
|  |  |  |
| **M5. Gene Expression**  **(211 nodes, 623 edges)** |  |  |
| EIF2 signaling | IPA | 1.2589E-75 |
| Regulation of eIF4 and p70S6K signaling | IPA | 3.1623E-21 |
| Phospholipase C signaling | IPA | 8.3176E-07 |
| Apoptosis signaling | IPA | 0.01348963 |
| Translational elongation | GO | 8.0121E-89 |
| Gene expression | GO | 1.2008E-39 |
| Cellular macromolecule biosynthetic process | GO | 1.8894E-38 |
| Cellular protein metabolic process | GO | 4.1766E-30 |
| Cellular metabolic process | GO | 2.8799E-19 |
| Leukocyte activation | GO | 9.2644E-05 |
|  |  |  |
| **M6. Metabolism**  **(189 nodes, 2,194 edges)** |  |  |
| 4-hydroxyproline degradation I | IPA | 0.01348963 |
| Proline biosynthesis I | IPA | 0.02630268 |
| Proline biosynthesis II (from arginine) | IPA | 0.03981072 |
| Arginine degradation VI (Arginase 2 pathway) | IPA | 0.03981072 |
| Fucose metabolic process | GO | 0.040412 |
| Carbohydrate metabolic process | GO | 0.20522 |
| Aminoglycan metabolic process | GO | 0.21765 |
| Hexose metabolic process | GO | 0.21765 |
| Monosaccharide catabolic process | GO | 0.21765 |
| Glycosaminoglycan metabolic process | GO | 0.36956 |

**Supplementary Table 4 – Upstream regulator analysis results for SRA vs HC mRNA.** Summary of predicted upstream microRNAs regulating SRA gene expression. Full upstream regulator analysis can be found here: 10.6084/m9.figshare.27968076

| **Upstream Regulator** | **Molecule Type** | **Predicted Activation State** | **Activation z-score** | **B-H corrected p-value** |
| --- | --- | --- | --- | --- |
| mir-183 | microRNA | Activated | 2.178 | 4.30E-09 |
| mir-96 | microRNA | Activated | 3.208 | 2.15E-08 |
| mir-21 (includes others) | microRNA | Inhibited | -3.214 | 2.45E-04 |
| mir-30 (includes others) | microRNA | Inhibited | -2.248 | 2.60E-04 |
| mir-34 (includes others) | microRNA | Inhibited | -2.538 | 3.58E-03 |
| mir-29 (includes others) | microRNA | Inhibited | -2.781 | 5.90E-03 |
| mir-506 (includes others) | microRNA | Inhibited | -2.401 | 7.07E-03 |
| mir-155 | microRNA | Inhibited | -2.557 | 1.24E-02 |
| let-7 (includes others) | microRNA | Inhibited | -2.236 | 1.98E-02 |
| mir-146 (includes others) | microRNA | Inhibited | -2.269 | 4.41E-02 |

**Supplementary Table 5 – Upstream regulator analysis results for SRA mRNA:miRNA target filter analysis.** Summary of predicted upstream microRNAs and transcription regulators. Full upstream regulator analysis can be found here: 10.6084/m9.figshare.27968238

| **Upstream Regulator** | **Molecule Type** | **Predicted Activation State** | **Activation z-score** | **p-value of overlap** |
| --- | --- | --- | --- | --- |
| ECSIT | transcription regulator | Inhibited | -2.176 | 9.59E-11 |
| ETV3 | transcription regulator | Inhibited | -2.53 | 2.04E-10 |
| NONO | transcription regulator | Activated | 3.578 | 1.22E-09 |
| IRF3 | transcription regulator | Activated | 2.885 | 1.69E-09 |
| STAT1 | transcription regulator | Activated | 2.082 | 6.25E-09 |
| TWIST1 | transcription regulator | Activated | 2.706 | 1.82E-08 |
| mir-183 | microRNA | Activated | 2.8 | 5.61E-08 |
| IRF7 | transcription regulator | Activated | 3.223 | 9.93E-08 |
| IRF1 | transcription regulator | Activated | 2.047 | 1.27E-07 |
| mir-96 | microRNA | Activated | 2.63 | 1.87E-07 |
| ETV6 | transcription regulator | Inhibited | -2.781 | 3.60E-07 |
| miR-182-5p (and other miRNAs w/seed UUGGCAA) | mature microRNA | Inhibited | -2.63 | 2.45E-06 |
| TRIM24 | transcription regulator | Inhibited | -2.804 | 1.18E-05 |
| STAT3 | transcription regulator | Inhibited | -2.341 | 1.30E-05 |
| KLF6 | transcription regulator | Inhibited | -2.789 | 6.06E-05 |
| FOXC1 | transcription regulator | Activated | 2.828 | 1.86E-04 |
| KLF2 | transcription regulator | Activated | 2.199 | 2.10E-04 |
| NFKB1 | transcription regulator | Inhibited | -2.689 | 2.28E-04 |
| ZFP36 | transcription regulator | Activated | 2.211 | 5.31E-04 |
| NFAT5 | transcription regulator | Inhibited | -2.63 | 8.05E-04 |
| MSC | transcription regulator | Activated | 2 | 3.01E-03 |
| GPS2 | transcription regulator | Activated | 2 | 3.75E-03 |
| STAT4 | transcription regulator | Inhibited | -2.03 | 5.95E-03 |
| WBP2 | transcription regulator | Activated | 2.236 | 9.29E-03 |
| TCF7L2 | transcription regulator | Inhibited | -2.4 | 2.55E-02 |
| SMARCA4 | transcription regulator | Inhibited | -2.236 | 2.68E-02 |
| NFIC | transcription regulator | Inhibited | -2 | 3.86E-02 |
